# Metabolic injury-induced NLRP3 inflammasome activation dampens phospholipid degradation

**DOI:** 10.1038/s41598-017-01994-9

**Published:** 2017-06-06

**Authors:** Elena Rampanelli, Evelyn Orsó, Peter Ochodnicky, Gerhard Liebisch, Pieter J. Bakker, Nike Claessen, Loes M. Butter, Marius A. van den Bergh Weerman, Sandrine Florquin, Gerd Schmitz, Jaklien C. Leemans

**Affiliations:** 1 Department of Pathology, Academic Medical Center Amsterdam, University of Amsterdam, Amsterdam, 1105 AZ The Netherlands; 2 Institute of Clinical Chemistry and Laboratory Medicine, University Hospital of Regensburg, Regensburg, 93053 Germany

## Abstract

The collateral effects of obesity/metabolic syndrome include inflammation and renal function decline. As renal disease in obesity can occur independently of hypertension and diabetes, other yet undefined causal pathological pathways must be present. Our study elucidate novel pathological pathways of metabolic renal injury through LDL-induced lipotoxicity and metainflammation. Our *in vitro* and *in vivo* analysis revealed a direct lipotoxic effect of metabolic overloading on tubular renal cells through a multifaceted mechanism that includes intralysosomal lipid amassing, lysosomal dysfunction, oxidative stress, and tubular dysfunction. The combination of these endogenous metabolic injuries culminated in the activation of the innate immune NLRP3 inflammasome complex. By inhibiting the sirtuin-1/LKB1/AMPK pathway, NLRP3 inflammasome dampened lipid breakdown, thereby worsening the LDL-induced intratubular phospholipid accumulation. Consequently, the presence of NLRP3 exacerbated tubular oxidative stress, mitochondrial damage and malabsorption during overnutrition. Altogether, our data demonstrate a causal link between LDL and tubular damage and the creation of a vicious cycle of excessive nutrients-NLRP3 activation-catabolism inhibition during metabolic kidney injury. Hence, this study strongly highlights the importance of renal epithelium in lipid handling and recognizes the role of NLRP3 as a central hub in metainflammation and immunometabolism in parenchymal non-immune cells.

## Introduction

Being a cluster of disorders (abdominal obesity, dyslipidemia, fasting hyperglycemia, hypertension), the metabolic syndrome (MetS) represents an escalating public-health problem. In the last years, a close association between MetS and renal dysfunction has emerged^1^; epidemiologic analyses showed that dyslipidemia and high BMI alone are independent risk factors for chronic kidney diseases (CKD) and the level of risk relates to the number of MetS components^2–4^. These observations strongly suggest a cause-effect relationship of MetS with CKD, which by themselves can be at the origin of vascular events, lipid abnormalities and insulin resistance, and hence cause of deteriorating metabolic condition^1^. Till now, the nature of the MetS-CKD association and the molecular pathomechanisms causally linking metabolic overloading to nephropathy remain elusive.

A key feature of metabolic disorders is chronic low-grade inflammation (metainflammation/metabolic inflammation). Among the many inflammatory mediators, the cytoplasmic innate Nod-like receptor NLRP3 has been implicated in promoting metabolic disorders. NLRP3 forms a large multimeric danger-sensing complex with the adaptor ASC and procaspase-1, leading to the autocatalytic activation of caspase-1 (CASP1) and, thus, cleavage of pro-IL-1β into its mature form. Several host-derived metabolites that are found elevated in obese/MetS individuals are held responsible for NLRP3 activation^5, 6^. A recent study from our group highlighted the importance of NLRP3 inflammasome in mediating diet-induced nephropathy^7^. In this study, mice fed a Western-type diet with a high-cholesterol content developed the typical traits of MetS together with nephropathy prominently characterized by tubular vacuolization with cholesterol and phospholipid accumulation in the proximal tubular epithelial cells (TEC). Interestingly, in *Nlrp3* knockout (KO) mice the pathological renal phenotype was significantly attenuated^7^, shedding light on the function of NLRP3 in regulating lipid metabolism and accumulation in TEC during metabolic stress/nutrient overloading. Nevertheless, the key mechanism explaining this phenotype has yet to be fully determined.

Due to the complexity of the MetS-renal disorder interrelationships, it is problematic to draw any definitive “cause-and-effect” conclusions. A part from hypertension and impaired glucose metabolism, other MetS aspects such as obesity and serum dyslipidemia may favor or provoke renal abnormalities and hence be considered new modifiable risk factors for CKD. Here, we aim to investigate in depth the potential causal link between dyslipidemia and impaired renal tubular function and to uncover the gap relating NLRP3 inflammasome activation and lipotoxicity in renal tubular cells. For this purpose, we extensively studied the impact of native or mildly oxidized LDL (n/oxLDL) on cultured TEC and of high-cholesterol diet (HCD) on kidneys from wild-type (WT) and *Nlrp3* KO mice on a Western-diet.

In relation to obesity and atherogenesis, special attention has been given to the biological activities of oxLDL, which in MetS originate from ROS-mediated LDL oxidation^8, 9^. For instance, oxLDL internalization by macrophages induces pro-inflammatory signaling and foam cell formation^10, 11^. Herein, we show that renal tubular epithelial cells resemble immune cells upon metabolic overload as witnessed by the induction of inflammatory pathways and intracellular lipid accumulation. Overall, this study reveals that LDL exerts detrimental effects on renal epithelial cells through a multifaceted mechanism that comprises the activation of the NLRP3 inflammasome by several endogenous triggers and the ability of the NLRP3/ASC/CASP1 complex to merge inflammatory and metabolic pathways. Therefore, targeting the metabolites-NLRP3-lipid catabolism axis seems a promising route in the fight against metabolic renal injury and presumably against diabesity in general.

## Results

### LDL instigates phospholipidosis and impairment of lysosomes in renal tubular cells

**Figure 1:**
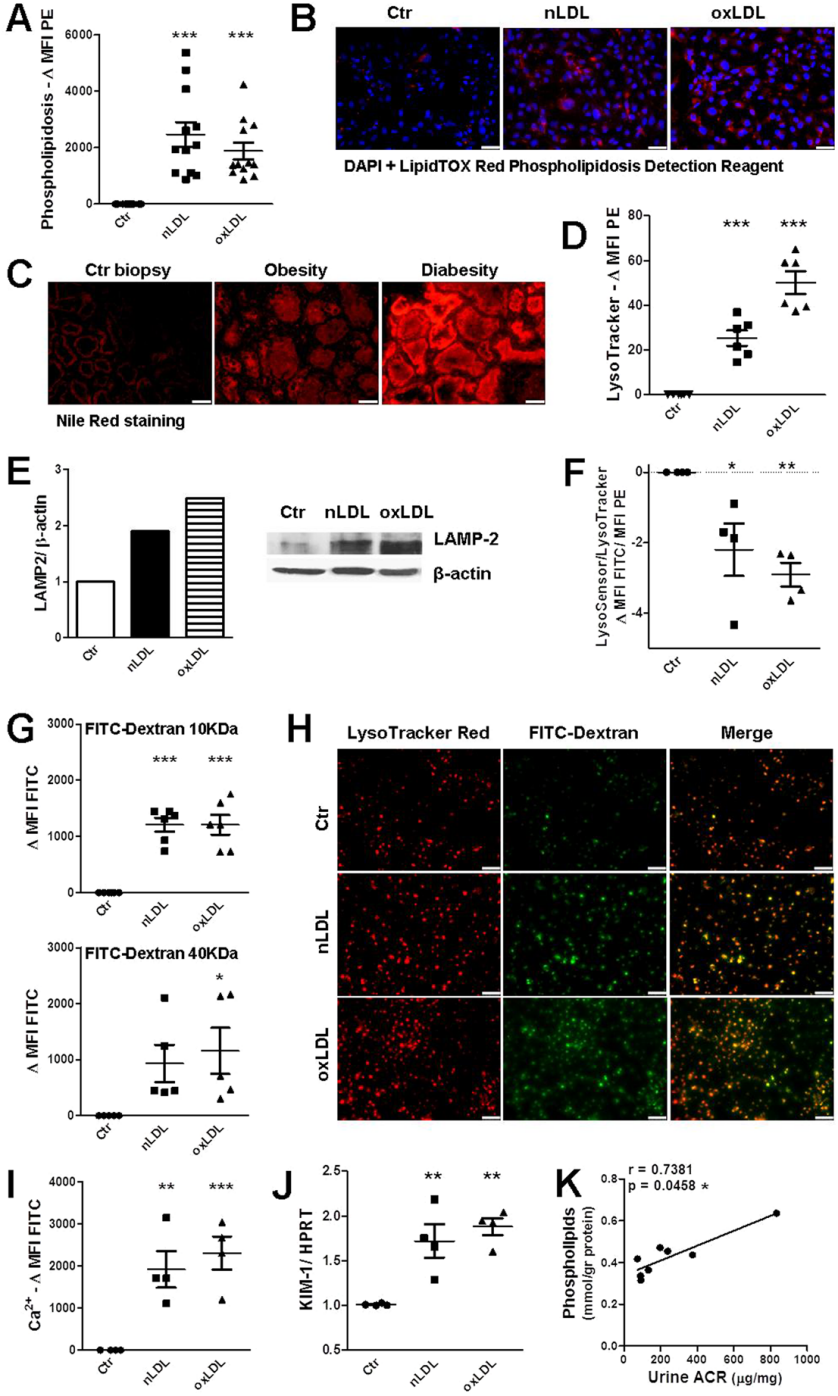
Lysosomal alterations induced by n/oxLDL loading in tubular cells. (**A**,**B**) Endolysosomal phospholipid accumulation detected by HSC LipidTox Red Phospholipidosis Detection Reagent at day 3. (**A**) Rise in phospholipidosis in HK2 tubular cells as compared to control (Ctr) (FC). MFIs of controls subtracted from the MFIs of n/oxLDL-treated cells. (**B**) Fluorescence microscopy images of control and LDL-loaded HK2 tubular cells to visualize phospholipidosis (red) and nuclei (blue, DAPI). Scale bar, 50 µm. (**C**) Nile Red staining of human frozen renal sections from individuals with no metabolic disorders, obesity with hyperlipidemia or with diabetes. Background staining digitally subtracted. Scale bar, 50 µm. (**D**–**F**) Lysosomal alterations upon n/oxLDL exposure for 3 days. (**D**) FC analysis of the PE fluorescence intensity emitted by LysoTracker Red-labelled endolysosomes in alive HK2 cells. MFIs normalized to controls by subtraction. (**E**) Western blot for LAMP-2 using HK2 cells lysates. Intensity normalized to β-actin loading control. Protein expression shown as fold increase compared to control equal to 1. (**F**) Changes in lysosomal acidity detected by pH-dependent LysoSensor Green probe. Dotplots showing FITC intensity in relation to the PE intensity of LysoTracker Red stained lysosomes (FC). (**G**) FC analysis of HK2 cells exposed to FITC-Dextran 10/40 KDa for 90 min after 3 days treatment with LPDS or n/oxLDL. Control group MFIs subtracted from MFIs of n/oxLDL groups. **(H)** 40 KDa FITC-Dextran (green) leakage from the lysosomes (red) of 3 days treated HK2 cells. Scale bar, 50 µm. **(I)** Cytoplasmic calcium detected by the Fluo-4-AM Ca^2+^ indicator (FC). Differences in FITC MFIs. (**J**) QPCR analysis for KIM-1 gene expression normalized for HPRT, relative to control. (**K**) Spearman rank correlation between urinary albumin to creatinine ratio (ACR, µg/mg) and renal phospholipid content (mmol/gr protein). WT mice fed a control or Western-diet; n = 8. (**A,﻿B,D﻿–J**) All assays performed after 3 days loading. Dots representing averages of independent experiments. Data shown as mean ± SEM; *P < 0.05, **P < 0.01, ***P < 0.001.

Since we found that high-cholesterol feeding causes cholesterol and phospholipid deposition within murine renal tubules and we know that LDL particle degradation initiates in lysosomes^6, 10^, we wondered whether n/oxLDL could provoke endolysosomal lipid accumulation (phospholipidosis) in diverse renal tubular epithelial cell lines (HK2, IM-PTEC, MDCK). Cultured TEC were treated with nLDL, oxLDL or lipoprotein-deficient serum (LPDS) as previously performed in other studies in different cell-types^12–15^. After 3 days treatment with 5 µg/ml n/oxLDL, tubular cells, labelled with HCS LipidTOX Red Phospholipidosis Detection Regent, displayed a prominent augmentation in the occurrence of phospholipidosis as assessed by flow cytometry (FC) and fluorescence microscopy (FM) (Figs 1A,B and S1A, S2A). Accordingly, kidney biopsies of patients with obesity and hypercholesterolemia or with diabesity exhibited increased phospholipid deposition within renal tubules (Fig. 1C). The phospholipid storage was accompanied by an expansion of the endolysosomal compartment in cultured TEC as evidenced by the increased intensity of LysoTracker Red staining and the enhanced expression of lysosome-associated membrane protein 2 (LAMP-2) (Fig. 1D,E). In contrast, cell staining with an acidity sensitive lysotropic Green fluorescent dye showed also a loss of lysosomal acidity, which is fundamental for a proper lysosomal “digestive function”^16^ (Figs 1F and S2B).

Next, we investigated whether lysosomal overloading would trigger lysosomal disruption. As revealed by the profound leakage of FITC-Dextran added to alive HK2 cells after 3 days n/oxLDL exposure, lysosomal membrane permeabilization (LMP) is part of the TEC intracellular alterations caused by metabolic overloading. Indeed, FC and FM analysis show respectively a FITC MFI (geometric mean fluorescence intensity) and a punctate green staining outside the LysoTracker Red-labelled lysosomes in n/oxLDL-treated TEC (Fig. 1G,H). The observed LMP is likely to account for the increased cytoplasmic calcium content detected by the Fluo-4-AM Ca^2+^ indicator since lysosomes contain high calcium concentration^16^ (Figs 1I and S2C). Finally, Western blotting showed that both native and oxidized LDL can strongly induce autophagy (Fig. S1B) but not endoplasmatic reticulum (ER)-stress response (Fig. S1C,D).

The n/oxLDL-mediated cellular toxicity was further evidenced by the upregulation of the kidney injury molecule-1 (KIM-1), an early marker of proximal tubule injury^17^ (Fig. 1J). In addition, the renal phospholipid concentration positively correlates with the degree of microalbuminuria, as index of diet-induced nephropathy^7^ (Fig. 1K), fortifying the concept of lipotoxicity-mediated renal damage/dysfunction.

### By fueling oxidative stress, LDL loading impairs the energetic status of tubular cells

**Figure 2:**
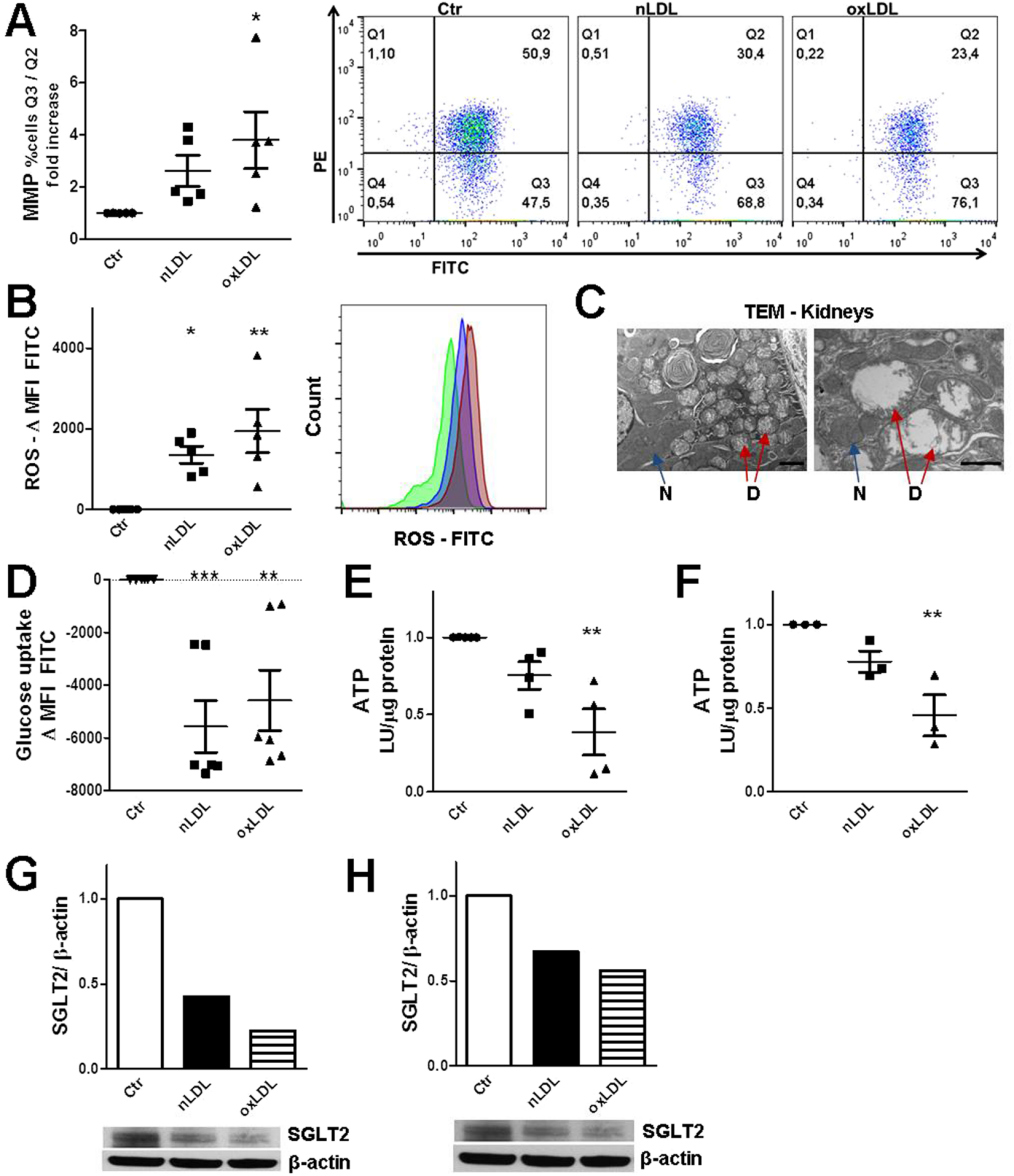
Mitochondrial damage and loss of tubular functional properties. (**A**) Mitochondria membrane permeabilization (MMP) assessed with MMP MITO-ID® Membrane Potential Detection cationic dye that fluoresces either green (as monomer in the cytosol) or orange (as aggregate in the mitochondria) depending upon membrane potential status. MMP indicated by increased ratio of %FITC + PE −/%FITC + PE + HK2 cells, quadrants Q3/Q2 of the scatterplot (FC). Data normalized to Ctr ( = 1). (**B**) Oxidative stress detected by Green fluorescent ROS Detection Reagent (FC). Data normalized by subtracting Ctr MFIs. Histogram plotting the FITC picks of HK2 cells exposed to LPDS (green), LDL (blue) and oxLDL (red). (**C**) Transmission electron microscope images of kidney sections derived from mice fed a Western-diet. Arrows indicate damaged (D) and normal (N) mitochondria. Scale bars, 2 and 1 µm. (**D**) Uptake of green fluorescent deoxyglucose analog (2-NBDG) by MDCK cells (FC). Control MFIs subtracted from n/oxLDL MFIs, negative values indicative of a reduction in 2-NBDG uptake. (**E**) Luminescent ATP Detection in MDCK and (**F**) HK2 cells. Data shown as ratio luminescence unit (LU)/µg protein of cell lysates divided by the Ctr values (Ctr = 1). (**G**,**H**) Westernblot for SGLT2 using protein lysates of HK2 cells after (**G**) 5 or (**H**) 3 days treatment. β-actin used as loading control. SGLT2 expression normalized to Ctr ( = 1). (**A**,**B**,**H**) Assays at day 3, (**D**–**G**) day 5. In dotplot graphs, each dot represents the average of one independent experiment; mean ± SEM; *P < 0.05, **P < 0.01, ***P < 0.001.

Since excessive cellular oxidative stress has been indicated as one of the defects in metabolic syndrome^18^, we questioned if kidney cells also harbor this “defect”. Both nLDL and oxLDL provoke mitochondrial damage and oxidative stress in TEC (Figs 2A,B and S2D) and tubular mitochondria are also susceptible to palmitate (Fig. S1E), a highly abundant fatty acid (FA) in high-fat diet (HFD)-associated hyperlipidemia^19^. Importantly, electron microscopy (EM) analysis of kidney specimens from mice subjected to HCD revealed morphological alterations in the mitochondria of proximal tubules, recapitulating the phenotype observed *in vitro* (Fig. 2C). In line with the above described phospholipid intratubular deposition, EM images of renal tubules also showed the development of many concentric multilamellar bodies (MLB), which arise from endolysosomal phospholipids accumulation likely due to impaired lysosomal hydrolysis^11^.

As tubular cells largely rely on mitochondrial oxidative metabolism-mediated ATP production to fulfil their absorptive functions^20^, lack of functional mitochondria can directly impair tubular reabsorption with inevitable systemic effects. Using the polarized tubular epithelial MDCK cells, we found that the glucose absorption declined after long exposure (5 days) to n/oxLDL (Fig. 2D). Concurrently, the intracellular ATP content drastically dropped in MDCK as well as HK2 tubular cells (Fig. 2E,F). In addition, 5/3-days n/oxLDL-treatment of HK2 cells profoundly reduced the expression of the sodium-glucose co-transporter 2 (SGLT2) (Fig. 2G,H), which imports glucose at the apical membrane using the downhill sodium gradient provided by the Na^+^/K^+^-ATPase pump located at the basolateral membrane^21^. Altogether, these data corroborate the hypothesis that hyperlipidemia alone directly mediates renal tubular dysfunction.

### Metabolic stress-driven NLRP3 inflammasome activation negatively regulates catabolic pathways by disrupting the SIRT1/LKB1/AMPK axis

**Figure 3:**
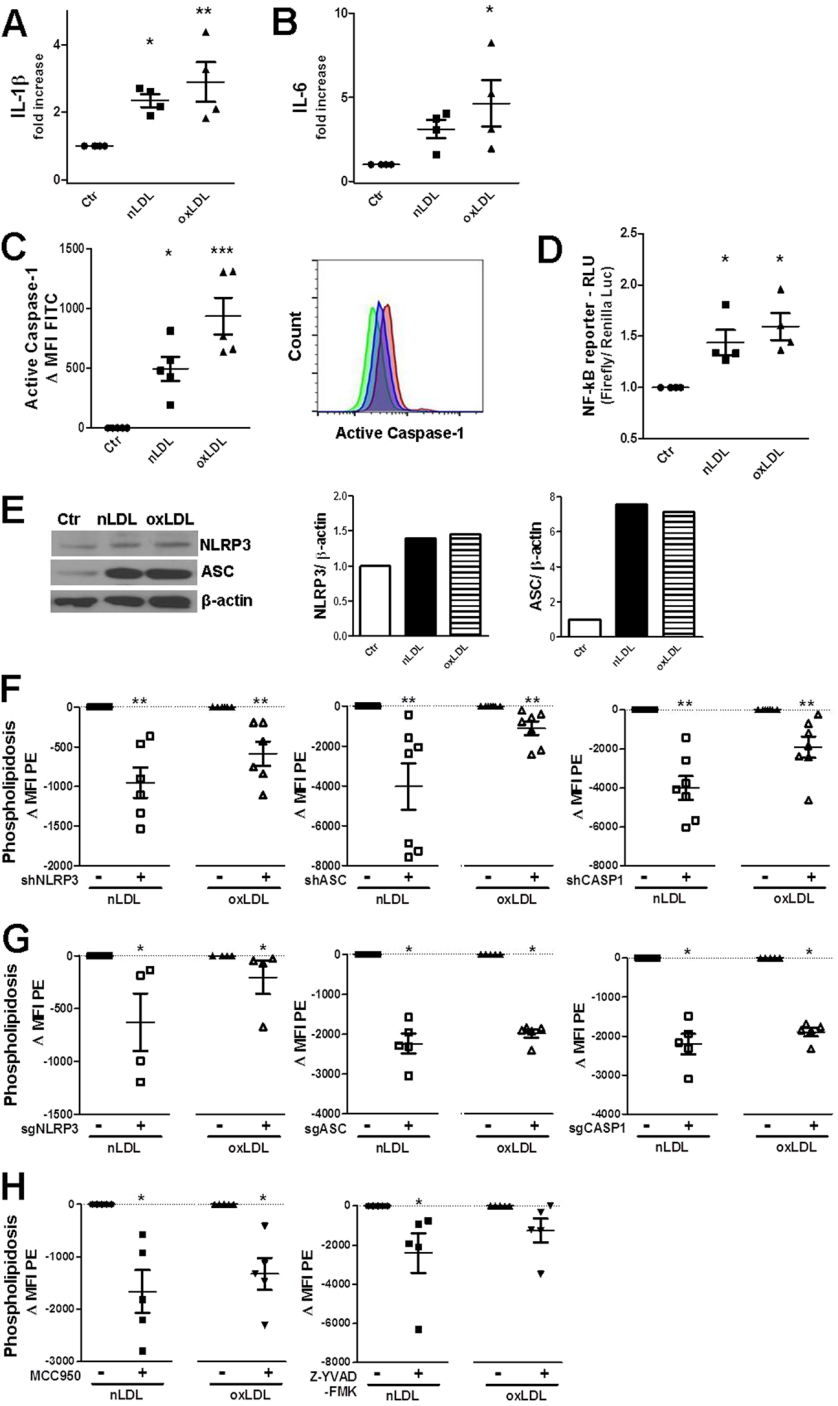
Activation of NLRP3 inflammasome during metabolic stress and regulation of phospholipidosis by the NLRP3/ASC/CASP1 complex. (**A**) Secretion of IL-1β and (**B**) IL-6 by IM-PTEC (ELISA). Cytokine concentrations (pg/ml) relative to Ctr equal to 1. (**C**) FC-based detection of active caspase-1 in HK2 cells using the green fluorescent FAM-YVAD-FMK. FITC MFI Ctr values subtracted from all MFIs values. FITC peaks shown in the right histogramplot: green (Ctr), blue (nLDL), red (oxLDL). (**D**) Dual-luciferase assay measuring the activities of NF-κB-driven Firefly luciferase (NF-κB reporter vector) and Renilla luciferase (control construct) in cell lysates of transfected HK2 cells. Values show relative luminescence units (RLU):Firefly/Renilla LU normalized to Ctr. (**E**) Westernblot for detection of NLRP3 and ASC in HK2 cells. Intensity normalized to β-actin loading control. Protein expression shown as fold increase compared to control equal to 1. (**F**) Phospholipidosis detection by FC after n/oxLDL loading in HK2 cells stably expressing lentiviral constructs encoding shRNA or (**G**) sgRNA targeting NLRP3, ASC, or CASP1 expression. Graphs showing the reduction in lipid storage in cells lacking full expression of the target gene in comparison to cells expressing the non-targeting sequence/empty vector. **(H)** Decline in fluorescence intensity emitted by LipidTox Red stained HK2 cells treated with NLRP3 (MCC950) and CASP1 (Z-YVAD-FMK) inhibitors (FC). **(A–D**,**F**,**G**) Dots representing averages of independent experiments; mean ± SEM; *P < 0.05, **P < 0.01, ***P < 0.001.

Chronic low-grade inflammation is an important contributor to the development of metabolic disorder. Since the establishment that nutrient overload coincides with peripheral tissue inflammation with M1 pro-inflammatory macrophages as important perpetuators of metabolic disease progression, much attention has been paid to the immunometabolism field and the metabolic programming/alterations of immune cells^5, 22^. We found that epithelial tubular cells resemble immune cells upon exposure to low-density lipoproteins; the secretion of the cytokines IL-1β and IL-6, the availability of activated caspase-1, the upregulation of NF-κB-driven luciferase, and the upregulation of NLRP3 and ASC testify the instigation of the NF-κB pathway and the activation of NLRP3/ASC/caspase-1 inflammasome (Figs 3A–E, S3A,B and S6A,B). In addition, active caspase-1 and ASC specks are visible by FM after LDL uptake (Fig. S3C,D). For comparison, THP-1 cells were treated with n/oxLDL and found to secrete IL-1β and IL-6, and to increase the protein expression of NLRP3 and ASC as in TEC (Fig. S3E,F). However,in THP-1 cells the basal expression of NLRP3 and ASC was higher and the n/oxLDL-induced ASC upregulation was milder than in kidney tubular cells. NLRP3 activation could be appointed to oxidative stress, mitochondrial damage, lysosomal leakage, accumulation of oxidized lipoproteins, palmitate or phospholipids, such as ceramide and cardiolipin^5, 6^.

In our model, the NLRP3 inflammasome complex not solely becomes activated by metabolic stress but it also influences LDL catabolism and determines the degree of LDL-induced endolysosomal phospholipidosis. Silencing of *NLRP3*, *ASC* or *CASP1* gene expression in HK2 cells by either shRNA or sgRNA significantly reduces phospholipidosis levels upon n/oxLDL treatment (Figs 3F,G and S4). Accordingly, specific inhibition of NLRP3 or caspase-1 with MCC950^23^ and Z-YVAD-FMK, respectively, also abates the degree of lipid storage after n/oxLDL uptake (Fig. 3H).

**Figure 4:**
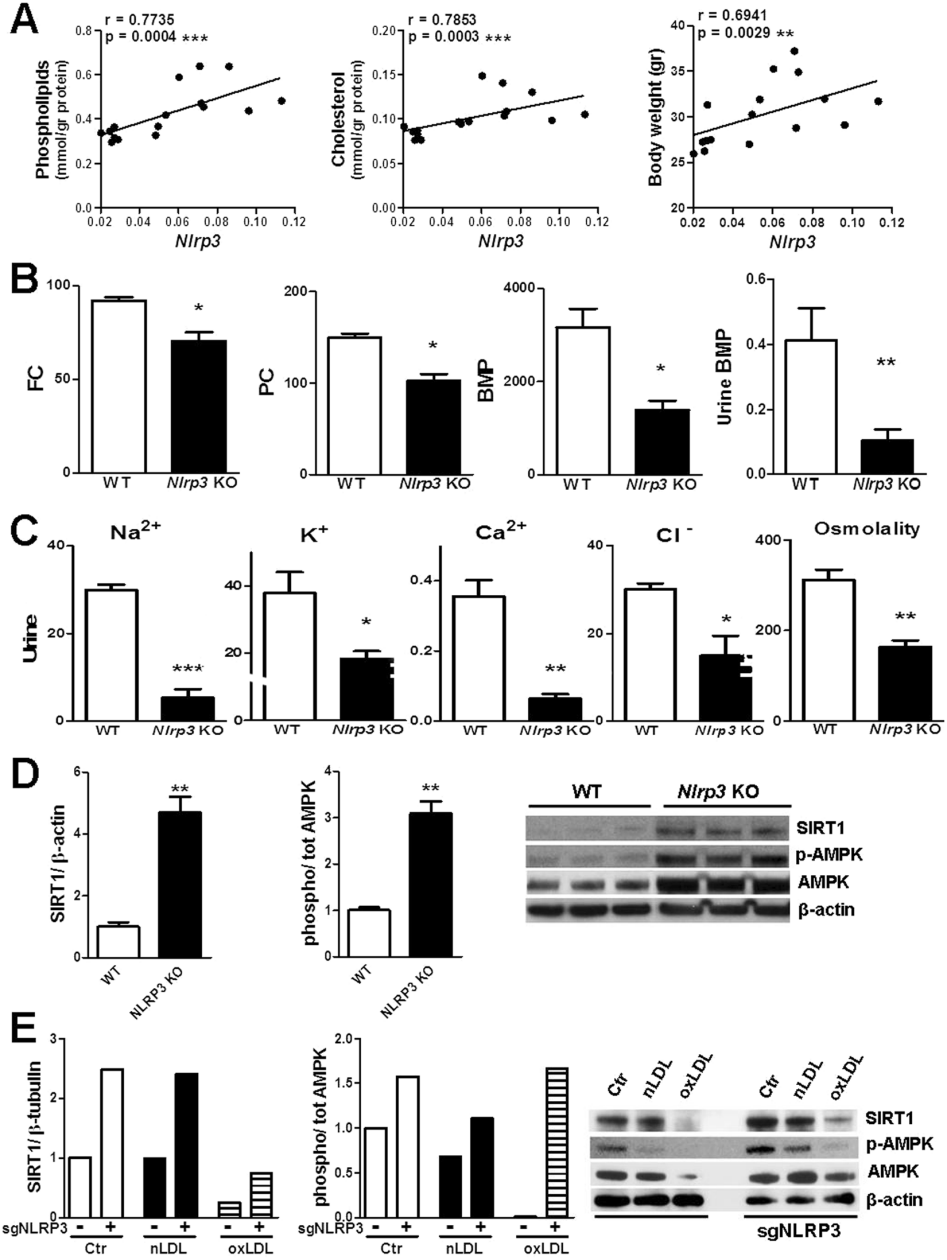
Differential lipid and protein expression in kidneys and cultured tubular cells lacking NLRP3. (**A**) Spearman rank correlation between *Nlrp3* renal gene expression and total renal phospholipid content, cholesterol (mmol/gr protein) and mice body weight (gr). WT mice fed a control or Western-diet; n = 16. (**B**) Mass spectrometry analysis of lipid content in kidney tissues and urine from WT and *Nlrp3* knockout mice on a Western-cholesterol enriched diet. Renal lipid species normalized for mg proteins: free-cholesterol (FC, nmol/mg protein), phosphatidylcholine (PC, nmol/mg protein), bis(mono)acylglycerol phosphate (BMP, pmol/mg protein); n = 4. Urine BMP (µmol/l) normalized for urine levels of creatinine (µmol/l); n = 8. (**C**) Urinary electrolyte concentrations (mmol/l) and urine osmolality (mOsm/kg) normalised for creatinine content (mmol/l); urine samples from WT and *Nlrp3* KO mice after 16 weeks of WD; n = 3. (**D**) Westernblot: SIRT1 expression and phosphorylation rate of AMPK in WT/*Nlrp3* KO kidneys of mice on a Western-diet; β-actin used as loading control. Data normalized to the values of WT kidneys; n = 3. (**E**) Westernblot showing the differences in full-length SIRT1 and AMPK activation rate between HK2 cells stably expressing or not sgRNA targeting *NLRP3* gene expression after 3 days treatment; β-actin used as loading control. Intensity values normalized to values of control cells transduced with empty vector. Data presented as mean ± SEM; *P < 0.05, **P < 0.01, ***P < 0.001.

Notably, renal *Nlrp3* expression correlates with total phospholipid and cholesterol stapling in WT murine kidneys and even with total body weight of WT mice (Fig. 4A). In line with the *in vitro* data, lipidomics on kidney tissues from HCD-fed mice revealed that the concentration of several lipid species was significantly lower in absence of NLRP3, namely free-cholesterol, the main phospholipid phosphatidylcholine, and the lysosomal phospholipid bis(monoacylglycero)phosphate (BMP)^11^ (Figs 4B and S5A). The latter was found at significantly lower concentration even in urine of NLRP3 knockout mice after HCD (Fig. 4B). Importantly for the translational potential of the present study, the urinary concentration of several electrolytes and osmolality were strikingly lower in urine specimens of *Nlrp3* KO mice after WD feeding (Fig. 4C), indicating a better tubular absorptive function in absence of NLRP3. Seeking for the underlying molecular function of NLRP3 during metabolic stress, we discovered that the expression and activation of AMPK (AMP-activated protein kinase), a central regulator of cell metabolism, and of its indirect activator sirtuin-1 (SIRT1) were more prominent in kidney tissues and cells lacking full NLRP3 expression (Fig. 4D,E). Sirtuin-1 was also found to be markedly expressed within renal tubules of NLRP3-deficient mice fed a HCD (Fig. S5B) and in isolated primary TEC from *Nlrp3* KO animals after LDL treatment as compared to WT tubular cells (Fig. S5C).

**Figure 5:**
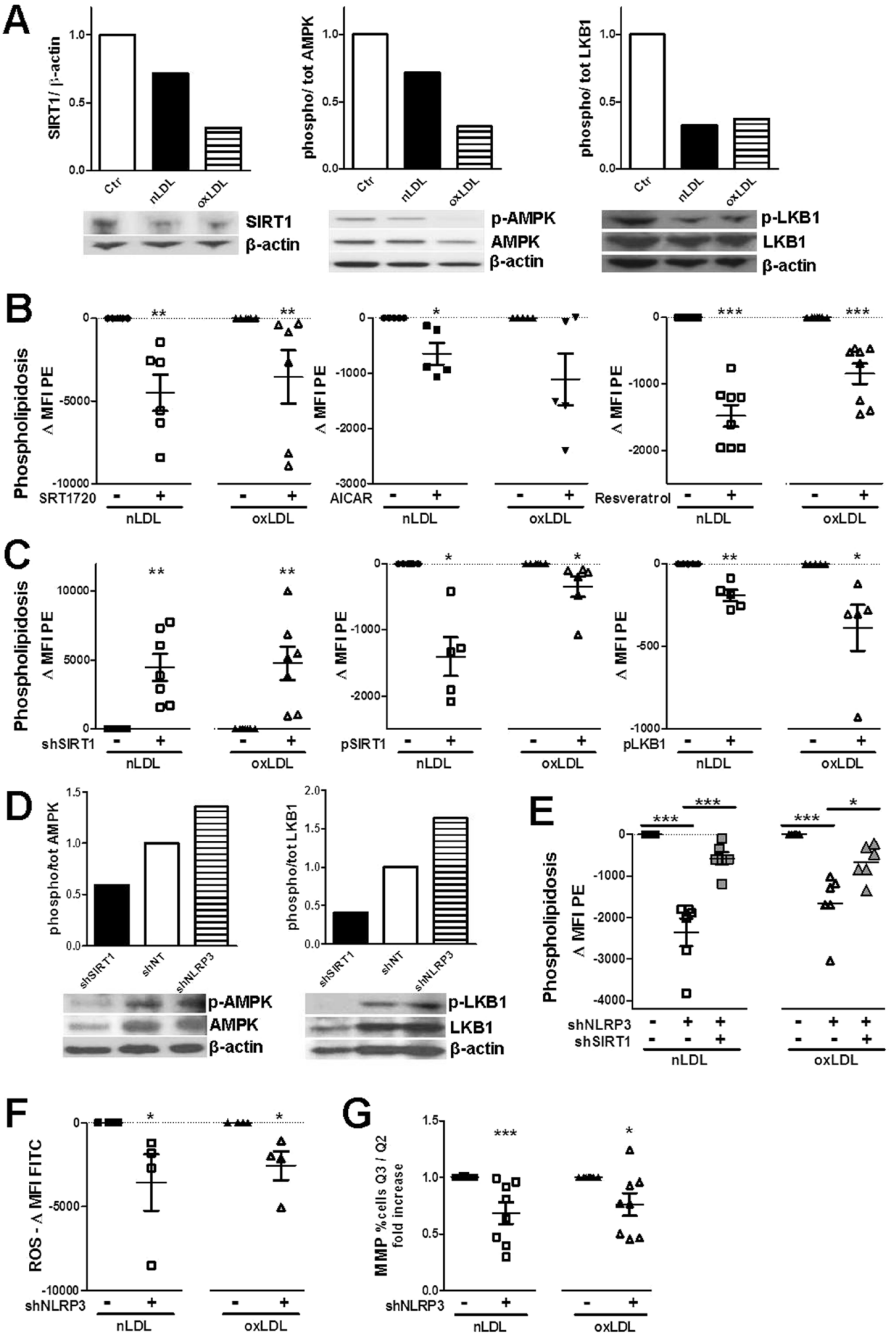
Negative regulation of SIRT1/LKB1/AMPK pathway by NLRP3/ASC/CASP1 immunocomplex. (**A**) Westernblots showing the expression of SIRT1, phosphorylated and total AMPK and LKB1 in HK2 cell lysates. Control value equal to 1 after normalization; β-actin used as loading control. (**B**) Use of SIRT1 activator (SRT1720) and AMPK activators (AICAR and resveratrol) to reduce phospolipidosis in respect to untreated cells after LDL loading (FC). Data shown as differences in MFI values. (**C**) Effects of SIRT1 knockdown (shRNA), overexpression (expression plasmid) of SIRT1 and LKB1 on the rate of endolysosomal phospholipid content in HK2 TEC in respect to their respective controls (non-targeting shRNA, empty vector). Data shown as differences in MFI values (FC). (**D**) Westernblot showing the activation rate of AMPK and LKB1 in LDL-loaded HK2 cells stably expressing shRNA targeting SIRT1/NLRP3 or non-targeting shRNA (shNT); β-actin used as loading control. Controls (shNT = 1) used for normalization. (**E**) Phospholipid storage in HK2 TEC expressing shRNA for *NLRP3*/*SIRT1* gene silencing (FC). MFI values of non-targeting shRNA expressing cells subtracted from all MFIs. (**F**) Oxidative stress detected by Green fluorescent ROS Detection Reagent (FC). Differences in MFI values of HK2 cells expressing shRNA targeting NLRP3 as compared to cells expressing shNT. (**F**) Mitochondria damage assessed with the MMP MITO-ID® assay; variations of MMP indicated by increased ratio of %FITC + PE-/%FITC + PE + HK2 cells (FC). Data normalized to values of cells expressing shNT ( = 1). (**B**,**C**,**E**,**F**,**G**) Dots representing averages of independent experiments. Mean ± SEM; *P < 0.05, **P < 0.01, ***P < 0.001.

Sirtuin-1 is a NAD^+^-dependent deacetylase and liver kinase B1 (LKB1) is one of its targets. Upon deacetylation, LKB1 can activate the catalytic subunit of the AMPK-complex^24^, thereby instigating catabolic pathways^25^. Importantly for our investigation, high-fat feeding was shown to induce proteolytic cleavage of SIRT1 by the NLRP3 inflammasome-activated caspase-1^26^. Our data highlight the importance of the SIRT1/LKB1/AMPK axis in renal lipid metabolism during nutrient overloading; n/oxLDL uptake diminished expression of SIRT1 and activation of AMPK and LKB1 in TEC (Fig. 5A). Accordingly, SRT1720-induced specific SIRT1 activation and AMPK activation by AICAR or resveratrol waned the phospholipidosis degree in n/oxLDL-treated TEC (Fig. 5B). Remarkably, resveratrol acted as a better deterrence of phospholipidosis; this can be attributed to its ability to simultaneous activate AMPK and SIRT1^27^. Modulations of SIRT1 expression in HK2 TEC by means of shRNA-mediated SIRT1 silencing or plasmid-mediated SIRT1 overexpression (Figure S4) led to opposite effects on lipid catabolism by increasing or decreasing, respectively, LDL-induced lipid accumulation. In accordance, LKB1 ectopic expression attenuated the phospholipidosis degree in n/oxLDL-loaded TEC (Fig. 5C).

As proof of the involvement of the NLRP3-CASP1/SIRT1/AMPK axis in the LDL-induced metabolic dysfunction, ablation of NLRP3 enhances the levels of active AMPK and LKB1 whereas knockdown of SIRT1 has the opposite effect upon LDL-overloading (Fig. 5D). Moreover, lack of both SIRT1 and NLRP3 abolishes the protective effect of NLRP3 silencing on n/oxLDL-triggered phospholipidosis (Fig. 5E), showing that NLRP3 targeting of sirtuin-1 mediates the inhibition of lipid breakdown.

Finally, the limitation of intralysosomal lipid storage resulted in a strong reduction of oxidative stress and defective mitochondria in NLRP3 knockdown cells (Fig. 5F,G).

**Figure 6:**
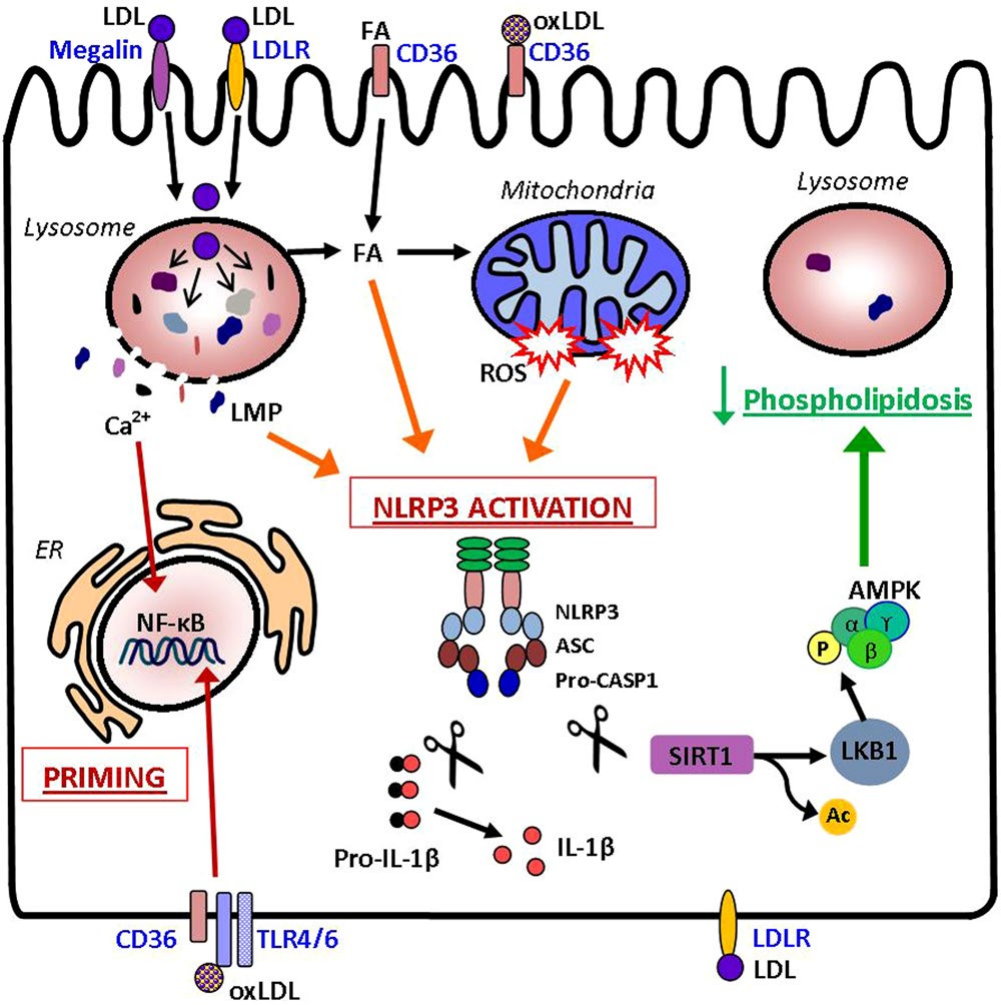
Lipotoxicity-induced NLRP3 inflammasome activation in TEC. After uptake by TEC via LDLR and megalin^36^, LDL particles are deliver to lysosomes for degradation releasing free-cholesterol and fatty acids (FA). FA can be directly uptaken by CD36 and boost mitochondria oxidation. oxLDL is recognized by CD36 and TLR4-TLR6 heterodimers. Excessive phagocytosis and impaired lysosomal hydrolysis of LDL particles cause phospholipidosis and lysosomal membrane permeabilization (LMP) allowing the release of lipase, cathepsins and Ca^2+^ into the cytoplasm. Lipoproteins and FA palmitate loading and calcium efflux can drive mitochondrial damage and ROS accumulation dropping ATP production and ATP-dependent functions. These effects occur in consequence to native and oxidized LDL uptake. NLRP3 inflammasome activation requires 2 signals: the 1^st^ for priming and the 2^nd^ for assembly. Priming is accomplished by NF-κB nuclear translocation induced by TLR4/6 and Ca^2+^ signaling. Lysosomal destabilization, mitochondrial damage, ROS, and saturated FA account for NLRP3 complex oligomerization and caspase-1 activation that in turn cleaves pro-IL-1β (maturation) and SIRT1 (inactivation). When activated, SIRT1 can deacetylate LKB1 allowing LKB1-mediated phosphorylation/activation of AMPK, an essential fuel gauge and promoter of mitochondrial β-oxidation and lipid catabolism. Thus, the metabolic stress-NLRP3/CASP1-SIRT1 cleavage pathway suppresses catabolic processes worsening the intralysosomal lipid deposition in TEC. Red arrows: priming; orange: NLRP3 activation; green: process counteracting lipotoxicity.

Figure 6 provides a graphical snapshot summarizing our data and known mechanisms explaining the lipotoxic injury and NLRP3 inflammasome activation in renal tubular cells.

## Discussion

Given the escalating prevalence of metabolic diseases and CKD among the global population and the mounting evidences of their harmful effects^1^, the demand for novel therapeutics is arising and hence the quest for novel studies exploring the causal relationship between these two complex diseases and potential common therapeutic targets.

In this study, we demonstrate that prolonged exposure of renal tubular epithelial cells to native and oxidized LDL triggers a cascade of events that comprise impairment of the lysosomal compartment, enhanced intralysosomal phospholipid content, oxidative stress and mitochondrial damage, reduced glucose uptake and ATP production. Altogether, our data prove the occurrence of metabolic damage and provide the till now missing mechanistic and causal link between MetS-hyperlipidemia and nephropathy. The described cascade of detrimental events caused by lipoproteins culminates in NLRP3 inflammasome oligomerization, a critical event in metabolically challenged kidney cells that is not merely a downstream effect of metabolic stress but an active mechanism controlling lipid catabolism through inactivation of the SIRT1/LKB1/AMPK cascade.

The first and more direct implications of LDL-uptake were detected in lysosomes of tubular cells; namely, lysosomal expansion with lipid amassing (phospholipidosis) and declined acidity. This lysosomal expansion could be a cellular mechanism to counteract the metabolic challenge through a cellular “lysosome rescue” program elegantly described by Sardiello *et al*.^28^ However, the herein reported drop in ATP and the free-cholesterol generation from LDL-cholesterol esters (CE) hydrolysis^11, 29^ contribute to a poorer v-ATPase proton pumping activity, accounting for diminished lysosomal acidity and, hence, diminished lipid breakdown. Thus, continuous overnutrition aggravates phospholipidosis in renal tubular cells due to the absence of fully functional acid lysosomal lipases. Remarkably, both native and oxidized LDL eventually induce damage and phospholipidosis in kidney tubular cells in a similar magnitude, despite being delivered to tubular lysosomes through different uptake routes: LDLR/megalin and pattern recognition/scavenger receptors for, respectively, nLDL and oxLDL^9, 29, 30^. These findings should shift the attention from the extensively studied oxLDL as pro-atherogenic agent^8, 9, 11^ to the relevance of nLDL as source of lysosomal disorder.

As documented by literature and our EM images, progressive phospholipid accumulation in adverse digestive conditions leads to the MLB formation within lysosomes. These structures together with the oxysterols derived from oxLDL degradation can be at the origin of lysosomal swelling and membrane destabilization^9, 11^. The consequential leakage of lysosomal content (proteases, lipases, Ca^2+^) into the cytoplasm is a trigger for inflammatory pathways^31–33^. We show a robust Dextran leakage from the lysosomes and cytoplasmic release of Ca^2+^ in metabolically stressed TEC. Lysosomal calcium efflux is held responsible for stabilizing the IL-1β transcript, instigating its release and triggering autophagy^31, 34, 35^. In lipid-loaded cells, autophagy may act as a protective mechanism to eliminate the disrupted lysosomes and to stop the leakage of their content in the cytoplasm^11, 16^. Our and others’ data highlight the importance of lysosomes in cell homeostasis^16^; aside from their “waste bag” functions, lysosomes take the “first-hit” in metabolic injury and their disruption/dysfunction adversely affects the functions of other cell organelles, such as mitochondria and autophagosomes.

Secondly, the present study demonstrates ROS accumulation and mitochondrial membrane permeability in TEC during metabolic overloading and mitochondrial damage in murine kidney tubules upon high-feeding state. These mitochondrial alterations may result from excessive Ca^2+^ uptake^32^ and excessive metabolic activities during overnutrition^19^. The increasing availability of FA in obese individuals is known to boost mitochondria metabolism^19^, and we show that palmitate elicits mitochondrial damage. In support of our data, an altered oxidative/antioxidant ratio is reported in MetS and the presence of oxidative stress markers increases proportionally to the number of MetS risk factors^36^. Mitochondrial damage can explain the reported ATP depletion in n/oxLDL-treated TEC. In line, the lessened glucose uptake can originate from the diminished SGLT2 expression as well as ATP depletion-mediated poorer activity of the Na^+^/K^+^-ATPase pump that provides the sodium gradient necessary for Na^2+^-glucose transport by SGLT2^21^. Importantly, Kang and colleagues linked defective FA oxidation, ATP depletion and neutral lipid deposition within TEC with kidney fibrosis in humans and mice^20^, and we previously showed that HCD induces fibrosis in murine kidneys^7^. Thus, LDL-mediated mitochondrial dysfunction might be at the internode between overnutrition and renal fibrosis, a hallmark of CKD. Altogether, the LDL-mediated lysosomal and mitochondrial dysfunction, the upregulation of the kidney injury marker KIM-1^17^ and the positive correlation between albuminuria and renal phospholipid content show that hyperlipidemia and tubular LDL-uptake are causes of renal lipotoxicity. Accordingly, HCD-feeding resulted in tubulointerstitial inflammation, ECM deposition and tubular malabsorption^7^.

In light of previous studies showing a strong and mutual relationship between inflammation and metabolism^5, 6^, and prominently the implication of the best characterized inflammasome NLRP3 in metabolic diseases^37–39^, including diet-induced nephropathy^7^, we pursued our investigation on the role of NLRP3 in metabolic injury-associated phospholipidosis within parenchymal kidney cells. Notably, this revealed a new function of NLRP3 in positively regulating the intracellular non-neutral lipid storage of parenchymal kidney cells upon metabolic challenge and the protective effect of lacking NLRP3 against tubular malabsorption in obese hypercholesterolemic mice. NLRP3 can act as a sensor of metabolic disturbance within cells. Indeed, in our study, uptake of native and oxidized LDL by TEC enhanced the activity of NF-κB and caspase-1 and the secretion of IL-1β indicating the occurrence of NLRP3 activation, which is classically viewed as a two-step process. The initial priming step consists of NFκB-mediated upregulation of NLRP3 and pro-IL-1β and post-translational modifications; the secondary NLRP3-specific activating signal leads to complex oligomerization and caspase-1 activation. Several pathways concomitantly participate to NLRP3 activation. Lysosome disturbances can account for the priming phase whereas the consequent cascade of intracellular alterations is responsible for the assembly phase in lipotoxic injury. For instance, a lysosomal calcium-calcineurin pathway was shown to regulate pro-IL-1β mRNA levels in primary macrophages^31^. Finally, the lysosome destabilization and leakage of the lysosomal protease cathepsin B culminate in NLPR3 inflammasome activation through a yet-undefined mechanism^32, 33^. Thus, through lysosomes, n/oxLDL loading can provide both signals for lipotoxic inflammasome activation. Nonetheless, oxLDL alone can provide both the priming and activating signals by promoting TLR4/6 signaling and causing nucleation of cholesterol in lysosomes after CD36-mediated uptake^10^. Moreover, the mitochondrial oxidative damage occurring in tubular cells upon metabolic overloading could be crucial for triggering NLRP3 immunocomplex oligomerization since mitochondria are central in NLRP3 inflammasome activation through a multifaceted mechanism (i.e. NLRP3 interaction with oxidized mitochondrial DNA, externalized mitochondrial cardiolipin, or with thioredoxin interacting protein)^39–42^. Finally, the activation phase in lipoprotein-stimulated TEC can also be instigated by saturated fatty acids (particularly palmitate) and ceramides contained in the LDL particles^38, 43^.

In this study, we show that silencing or inhibiting one of the components of the NLRP3 inflammasome has a profound inhibitory effect on phospholipidosis and, remarkably, the expression of sirtuin-1 is necessary for the NLRP3-mediated inhibition of lipid digestion. Correspondingly, kidney cells lacking NLRP3 display higher activation rate of AMPK and elevated expression of uncleaved full-length SIRT1. This can be explained by the study of Chalkiadak and Guarent^26^ showing that HFD induces cleavage of sirtuin-1 by inflammasome-associated caspase-1. The NAD^+^-dependent deacetylase sirtuin-1 promotes mitochondria biogenesis and metabolism, and lipid catabolism *via* a multifaceted mechanism that involves the direct deacetylation of several transcription factors and of LKB1^24, 44, 45^. The latter was found to be more active in cells lacking NLRP3 and it can directly phosphorylate and hence activate the energy sensor AMPK-complex abundantly expressed in kidneys^25^. AMPK instigates catabolic pathways, by enhancing glycolysis, fatty acid oxidation and mitochondrial biogenesis, and concurrently inhibits anabolic pathways by suppressing the activities of the rate-limiting step enzymes in cholesterol and fatty acid synthesis. By potentiating mitochondrial metabolism and hence increasing NAD^+^ levels, AMPK favors SIRT1 activity; this explains many of the convergent biological effects of AMPK and SIRT1 on energy metabolism^25, 46^. Indeed, in our model activation of AMPK or SIRT1 dampens the lipid storage within TEC. These observations are supported by previous studies showing that administration of AMPK or SIRT1 activators to mice given a prevent systemic and tissue-specific HFD-mediated metabolic disturbances/pathologies^47–49^. Notably, the inhibitory effects of *ASC* or *CASP1* silencing on intralysosomal lipid amassing are more pronounced in respect to the effect of *NLRP3* silencing; this phenomenon arises the question whether other ASC-dependent inflammasomes (NLRP1/NLRC4/AIM2) are indirectly implicated in metabolic pathways or whether other caspase-1 targets are at play^32^, such as the adipogenic transcription factor PPARϒ (peroxisome proliferator-activated receptor ϒ). Indeed, He *et al*. demonstrated that selective inhibition of caspase-1 with Z-YVAD-FMK blocks PPARϒ cleavage in cultured adipocytes^50^. Strikingly, lack of NLRP3 not solely counteract phospholipidosis but (as a likely consequence) also oxidative stress and mitochondria damage during lipoprotein overload and *in vivo* conserve tubular function upon Western-diet feeding.

In support of the *in vitro* data, we found significantly less lipid content in kidneys from mice on a Western-diet lacking NLRP3. Among the lipids analyzed, the glycerophospholipid BMP merits particular attention being exclusively found in the inner membranes of lysosomes and late endosomes and regarded as a marker for lysosomal storage disease^8^. The lesser BMP concentration in kidneys of *Nlrp3* KO mice may indicate a lower content of lipid-engulfed lysosomes incapable of unloading or depredating their cargo. The similar pattern of BMP content in kidneys and urine of WT and *Nlrp3* KO mice could indicate that urinary BMP might become a non-invasive marker of diet-induced renal lipotoxicity with future investigations.

## Conclusions

Altogether, our results show for the first time a direct lipotoxic effect of n/oxLDL on renal tubular cells and validate NLRP3 involvement in the onset and progression of metabolic disorders and suggest the potential of specifically targeting NLRP3 as a therapeutic approach to counteract or attenuate obesity/MetS-driven renal pathology. Precluding inflammasome oligomerization and caspase-1 autoactivation would prevent the downregulation of catabolic pathways and the increased intracellular lipid accumulation as well as block the extracellular effects of IL-1β in driving inflammation and metabolic disturbances.

Therefore, our observations provide a rationale for intervention studies that aim to verify whether blocking NLPR3 inflammasome activation can effectively prevent obesity/MetS-induced damage or abnormalities in the kidneys but also in other organs.

## Methods

Experimental procedures are described in great detail in *Supplementary Information*.

LDL particles were isolated from plasma from multiple healthy plasma donors by gradient ultracentrifugations^12, 13^. Human n/oxLDL was used at 5 µg/ml. Mild oxidation of LDL was achieved with 5 µM CuSO_4_. Lipoproteins and compounds were added at day 0 and every second day: 100 µM MCC950, 1 µM Z-YVAD-FMK, 20 mM AICAR, 50 µM Resveratrol, or 1 µM SRT1720. Stable knockdown cells were generated by transduction with lentiviral particles harbouring sh/sgRNA and puromycin selection.

For detection of alterations in lysosomal and mitochondrial organelles and tubular absorption, the following reagents were used: HCS LipidTOX™ Red phospholipidosis detection reagent (1:2000, Thermo Fisher Scientific), 50 nM LysoTracker Red DND-99 (Thermo Fisher Scientific), 1 µM pH-sensitive LysoSensor Green DND-189 (Thermo Fisher Scientific), 50 µg/ml FITC-Dextran 10/40 KDa (Sigma-Aldrich), 1 µM Fluo-4-AM (Invitrogen), MITO-ID® Membrane Potential Detection Kit (Enzo Life Sciences) and Oxidative Stress Detection Reagent (Enzo Life Sciences), 5 mM 2-NBDG (Cayman Chemical), 0,1 µg/ml Nile Red (Sigma Aldrich).

Stainings were visualized on Leica DM5000B microscope(Leica-microsystems) for FM, and on FACSCalibur, FACSCanto II or LSRFortessa (BD Biosciences) for FC (data analyzed with FlowJo software).

The following antibodies were used for WB, IF, IHC: anti-LAMP-2-FITC (LifeSpan BioSciences), anti-SIRT1 (Abcam), anti-phospho- and total AMPK, LKB1 (Cell Signaling Technologies), anti-β-actin (Abcam), HRP-conjugated secondary antibodies (DAKO), anti-ASC (Enzo Life Sciences), anti-rabbit IgG Alexa Fluor 488 (Invitrogen).

LDH release, intracellular ATP, cytokine secretion, NF-κB signaling, gene expression were quantified using LDH-Citotoxicity Assay Kit (BioVision), ViaLight™ Plus BioAssay Kit (Millipore), ELISAs (R&D Systems), Dual-Luciferase® Reporter Assay System (Promega), SYBR Green-SensiMix (Bioline) and LightCycler® 480 (Roche).

Lipids were isolated by chloroform/methanol extraction and quantified by direct flow injection electrospray ionization tandem mass spectrometry (ESI-MS/MS); sphingolipids were extracted by the butanolic extraction and analyzed by liquid chromatography-tandem mass spectrometry (LC-MS/MS) protocol.

Statistics was performed using One-Way ANOVA and Dunnett’s tests, Mann-Whitney U test, or Spearman rank correlation; P < 0.05 was considered to be significant. Data presented as mean and standard error of the mean (SEM). In dotplots, each dot represents the average of one independent experiment.

## Electronic supplementary material

Supplementary Figure and Methods
