# Supplementary material for: Metabolic injury-induced NLRP3 inflammasome activation dampens phospholipid degradation
Source: Sci Rep. 2017 Jun 6;7:2861. doi: 10.1038/s41598-017-01994-9 (PMC5460122; doi:10.1038/s41598-017-01994-9)
Supplement: Supplementary file 1 — Supplementary Figure and Methods [file 41598_2017_1994_MOESM1_ESM.pdf]

Metabolic injury-induced NLRP3 inflammasome activation dampens phospholipid degradation

Elena Rampanelli[1,2,*], Evelyn Orsó[2], Peter Ochodnicky[1], Gerhard Liebisch[2], Pieter J. Bakker[1], Nike Claessen[1], Loes M. Butter[1], Marius A. van den Bergh Weerman[1], Sandrine Florquin[1,3], Gerd Schmitz[2], Jaklien C. Leemans[1]

[1]Department of Pathology, Academic Medical Center Amsterdam, University of Amsterdam, Amsterdam, 1105 AZ, The Netherlands
[2]Institute of Clinical Chemistry and Laboratory Medicine, University Hospital of Regensburg, Regensburg, 93053, Germany
[3]Department of Pathology, Radboud University Nijmegen Medical Center, Nijmegen, 6525 HP, The Netherlands

[*]**Corresponding author**: Elena Rampanelli, PhD
Department of Pathology, Rm L2-111
Academic Medical Center, University of Amsterdam
Meibergdreef 9, 1105 AZ Amsterdam, The Netherlands
Tel: +31 20 566 65715
Fax: +31 20 566 9523
e-mail: e.rampanelli@amc.uva.nl

Supplementary Information:

Supplementary Figure and Figure legends & Supplementary Methods

Supplementary Figures and Figure legends

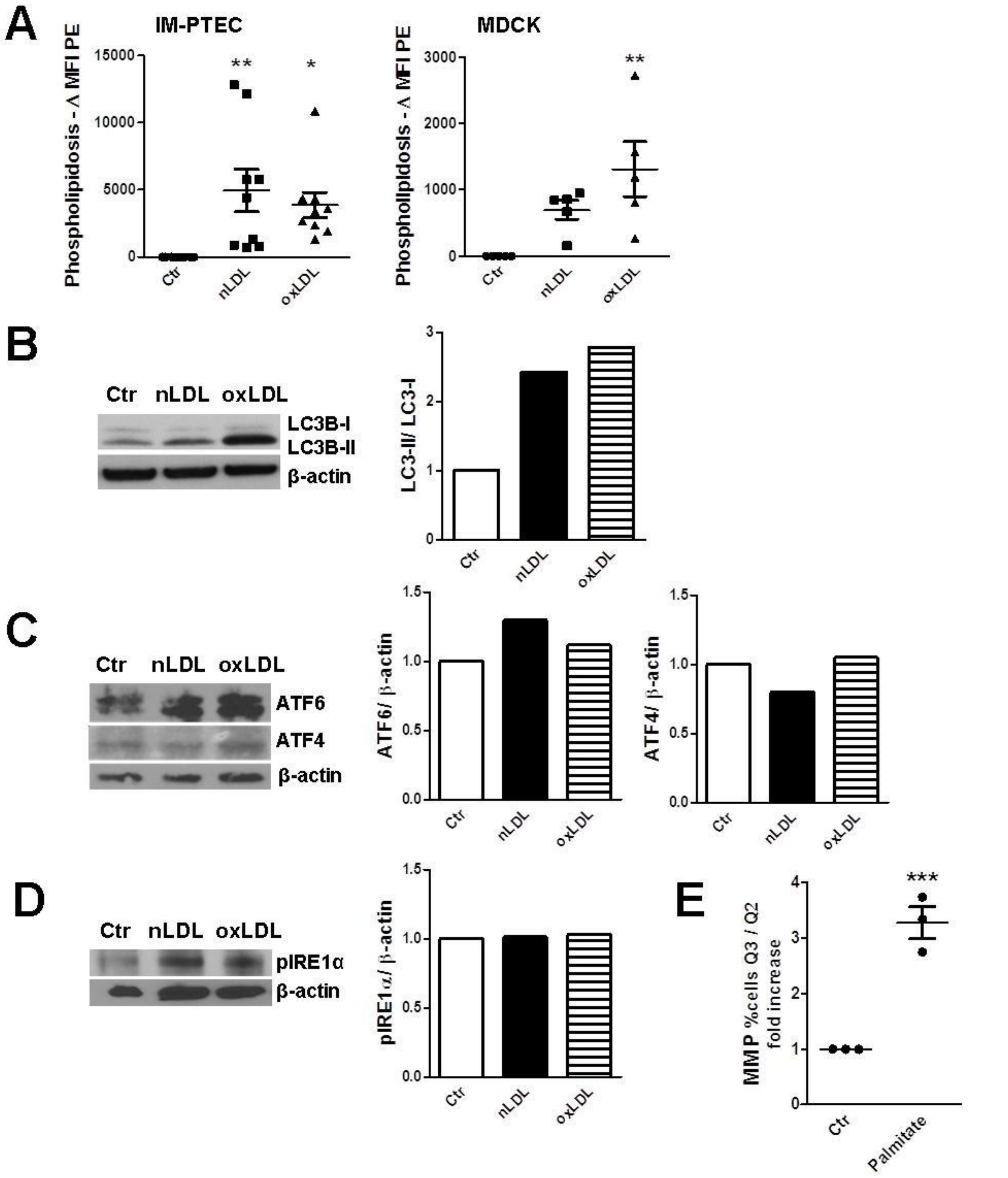

Figure S1. (A) Rise in phospholipidosis in immortalized IM-PTEC and MDCK tubular cells as compared to controls (FC). MFIs of controls subtracted from the MFIs of n/oxLDL-treated cells. **(B-D)** Westernblots for detection of **(B)** LC3B, **(C)** ATF6, ATF4 and **(D)** phospho-IRE1α. β-actin used as loading control. Data normalized to the values of controls. **(E)** Mitochondria damage: MMP induced by palmitate loading compared to control equal to 1. FC analysis of MITO-ID® stained HK2 cells. Data shown as mean ± SEM; *P<0.05, **P<0.01, ***P<0.001.

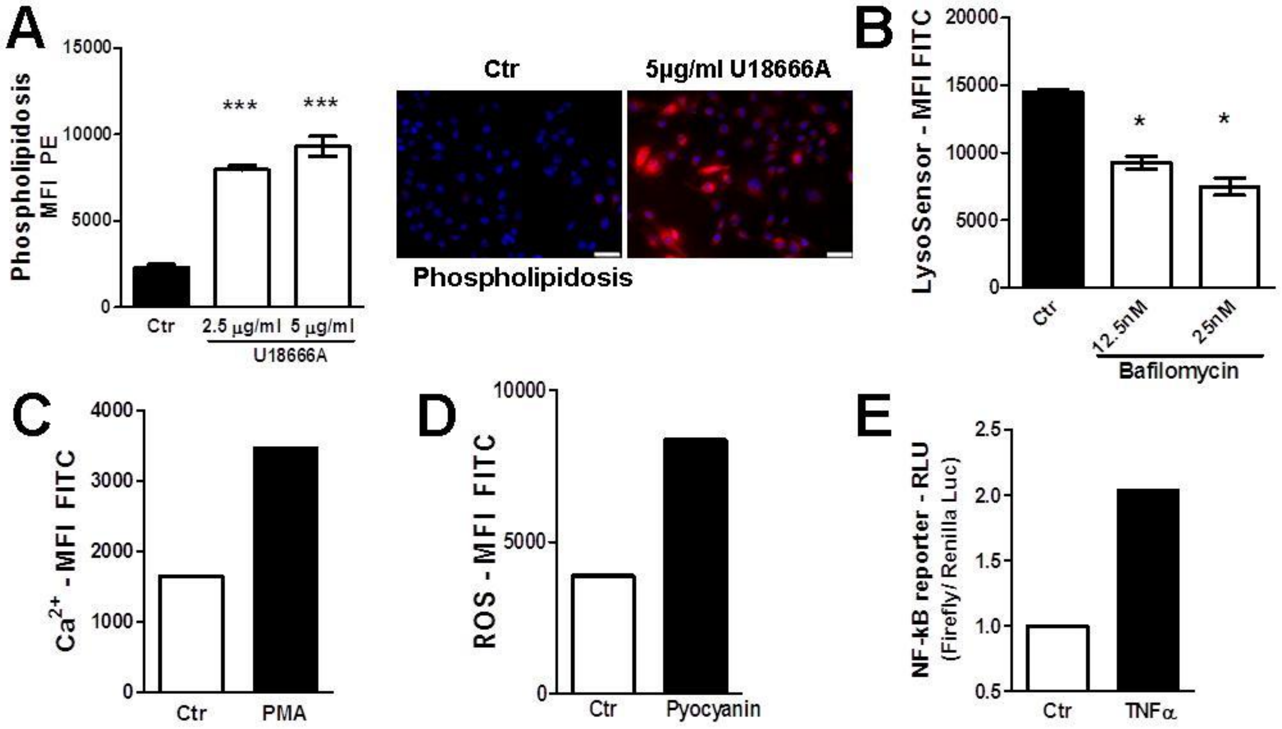

Figure S2. Positive controls. **(A-D)** Flow cytometry analysis of HK2 cells. **(A)** Phospholipidosis induction with increasing concentration of 5µg/ml U18666A, 24 hours. Fluorescent microscopy images to visualize phospholipidosis (red) and DAPI-stained nuclei (blue). Scale bar, 50 µm. **(B)** Decreased lysosomal acidity with rising concentration of Bafilomycin A1 (2 hours exposure), detected by LysoSensor Green probe. **(C)** Calcium accumulation induced by 30 minutes treatment with 2µM PMA, Fluo-4-AM Ca2+ indicator. **(D)** Oxidative stress induced by 30 minutes treatment with 100µM Pyocyanin, Green ROS Detection probe. **(E)** Dual-luciferase reporter assay: relative luciferase activities of NF-κB-driven Firefly and Renilla luciferase in transfected HK2 cells stimulated or not for 24 hours with 10ng/ml TNFα. Relative luminescence units (RLU): Firefly/Renilla LU; control equal to 1. Statistical analysis done with t-test. Data represented as mean ± SEM; *P<0.05, ***P<0.001.

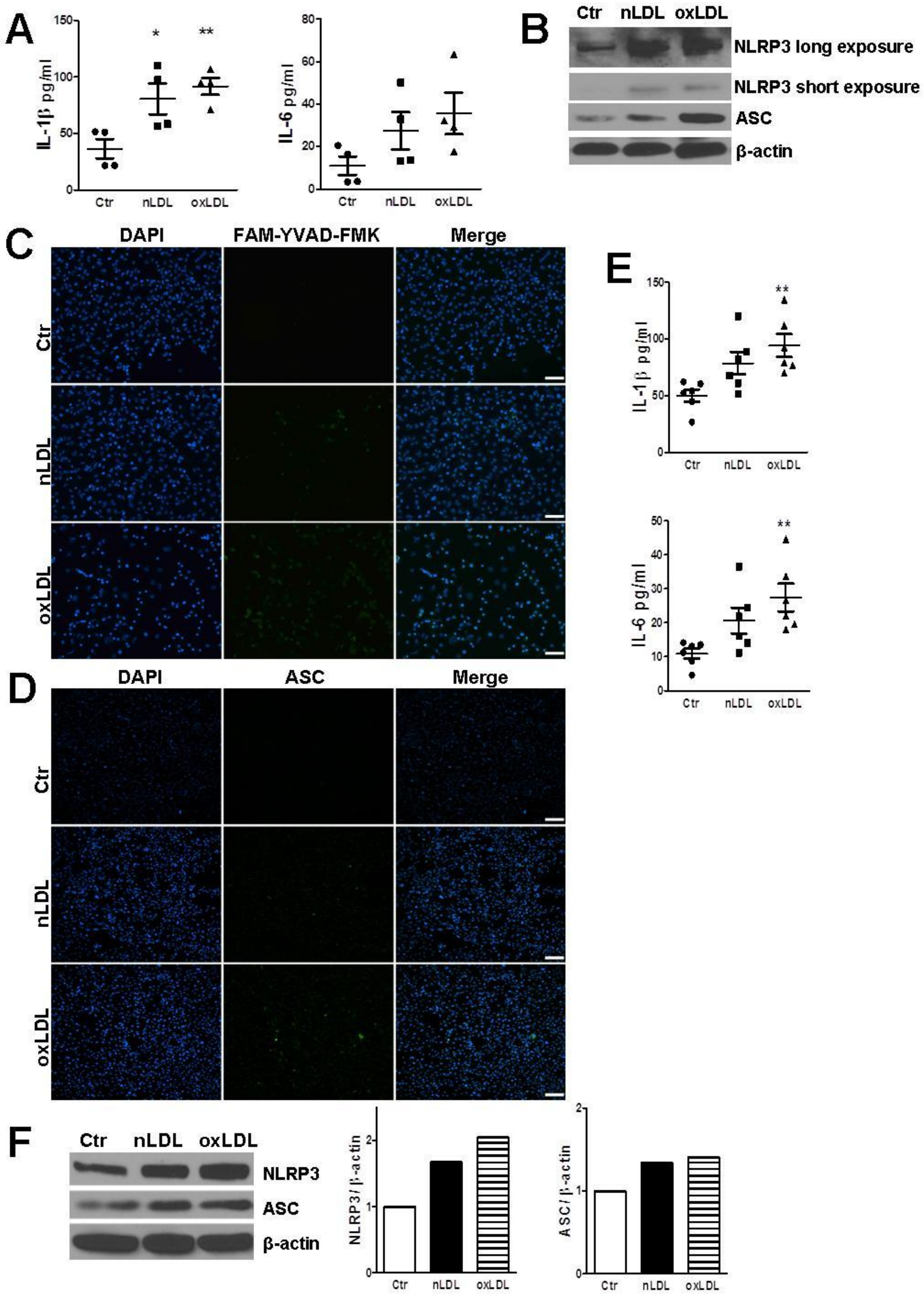
A
IL-1β pg/ml
IL-6 pg/ml
Ctr
nLDL
oxLDL
B
Ctr nLDL oxLDL
NLRP3 long exposure
NLRP3 short exposure
ASC
β-actin
C
DAPI
FAM-YVAD-FMK
Merge
Ctr
nLDL
oxLDL
D
DAPI
ASC
Merge
E
F
NLRP3
ASC
β-actin
NLRP3 / β-actin
ASC / β-actin

Figure S3. NLRP3 inflammasome activation. (A) ELISA for detection of IL-1β and IL-6 in supernatant of IM-PTEC; cytokine concentrations expressed in pg/ml, relative to Figures 3A and 3B. Dots representing averages of independent experiments. **(B)** Westernblot for NLRP3, ASC and β-actin using HK2 cell lysates. **(C,D)** Fluorescence microscopy images of control and n/oxLDL-loaded HK2 tubular cells to visualize **(C)** active Caspase-1 (FAM-YVAD-FMK) and **(D)** ASC (green) and nuclei (blue, DAPI). Scale bar, 50 µm. **(E)** ELISA for detection of IL-1β and IL-6 (pg/ml) in supernatant of THP-1 cells. **(F)** Westernblot for NLRP3 and ASC using THP-1 cell lysates. Intensity normalized to β-actin loading control. Protein expression shown as fold increase compared to control equal to 1. Data represented as mean ± SEM; *P<0.05, **P<0.01.

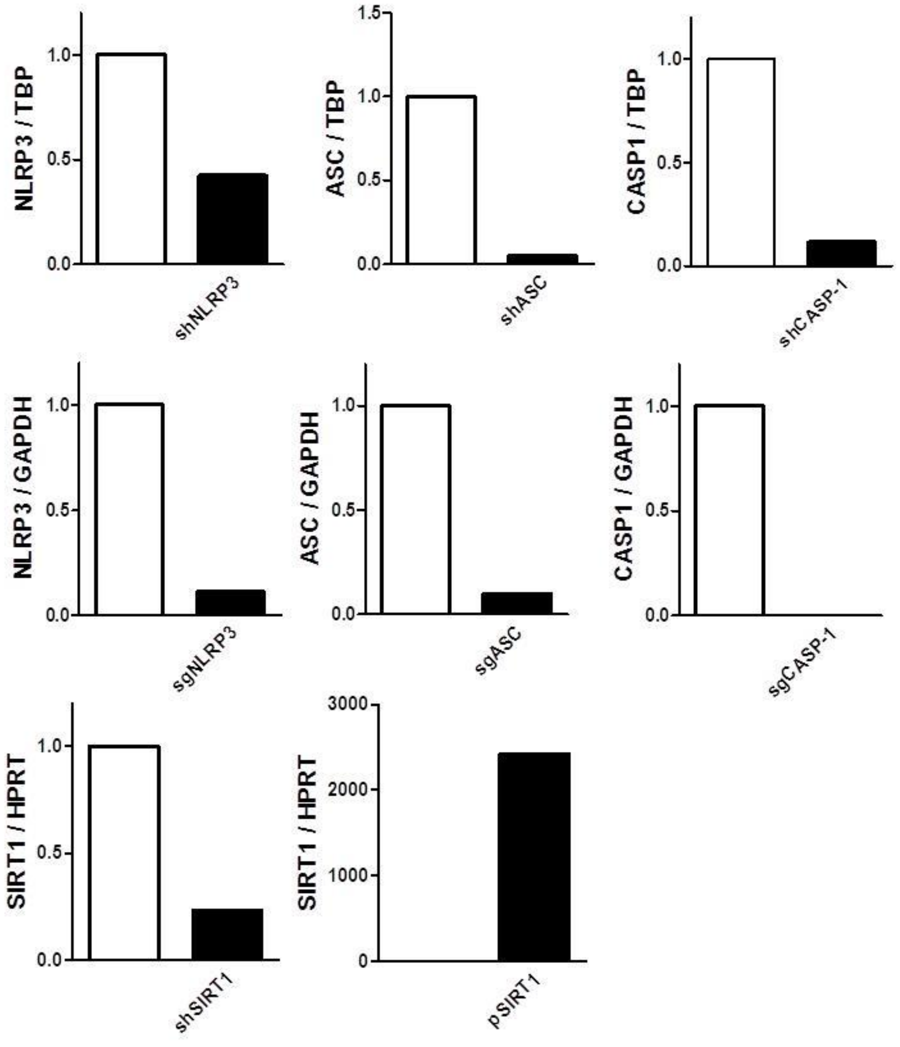

Figure S4. QPCR analysis. Gene expression of sh/sgRNA targeted genes in HK2 cells: *NLRP3, ASC, CASP1, SIRT1*. Gene expression of *SIRT1* in HK2 cells transfected with pSIRT1.

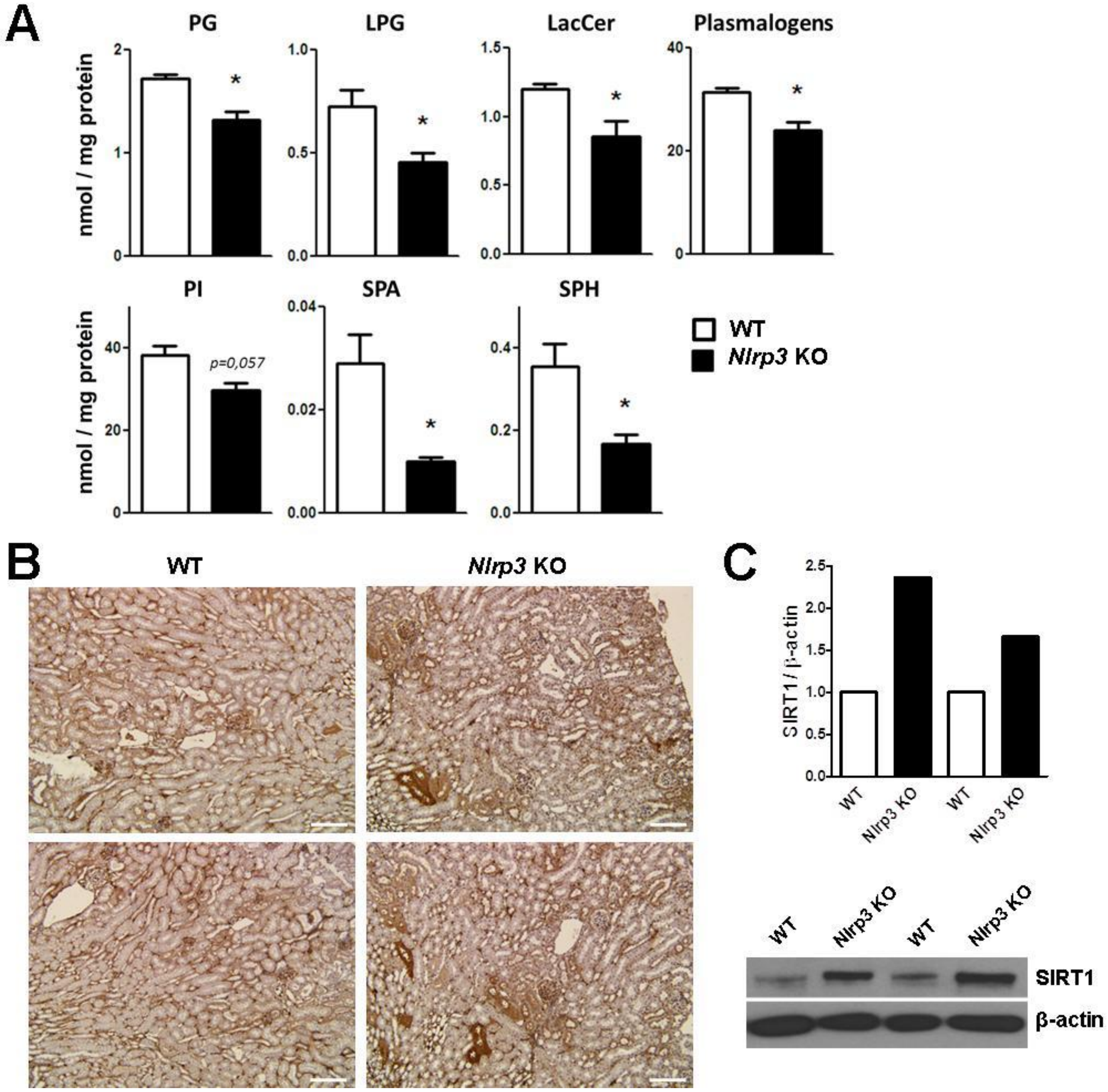

Figure S5. (A) Mass spectrometry analysis of kidney tissues from WT and *Nlrp3* KO mice fed a Western-diet. Lipid levels (nmol) normalized for mg protein. Lipid species detected: phosphatidylglycerol (PG), lysophosphatidylglycerol (LPG), lactosylceramide (LacCer), plasmalogens, phosphatidylinositol (PI), sphinganine (SPA), sphingosine (SPH); n=4. Data shown as mean ± SEM; *P<0.05. **(B)** Immunohistochemistry staining for Sirtuin-1 on paraffin sections of kidneys from WT and *Nlrp3* KO mice fed a HCD. Scale bar, 50 µm. **(C)** Expression of SIRT1 assessed by westernblot using cell lysates of primary TEC isolated from WT and *Nlrp3* KO kidneys and exposed to LDL for 3 days. β-actin used as loading control; values normalized to control equal to 1.

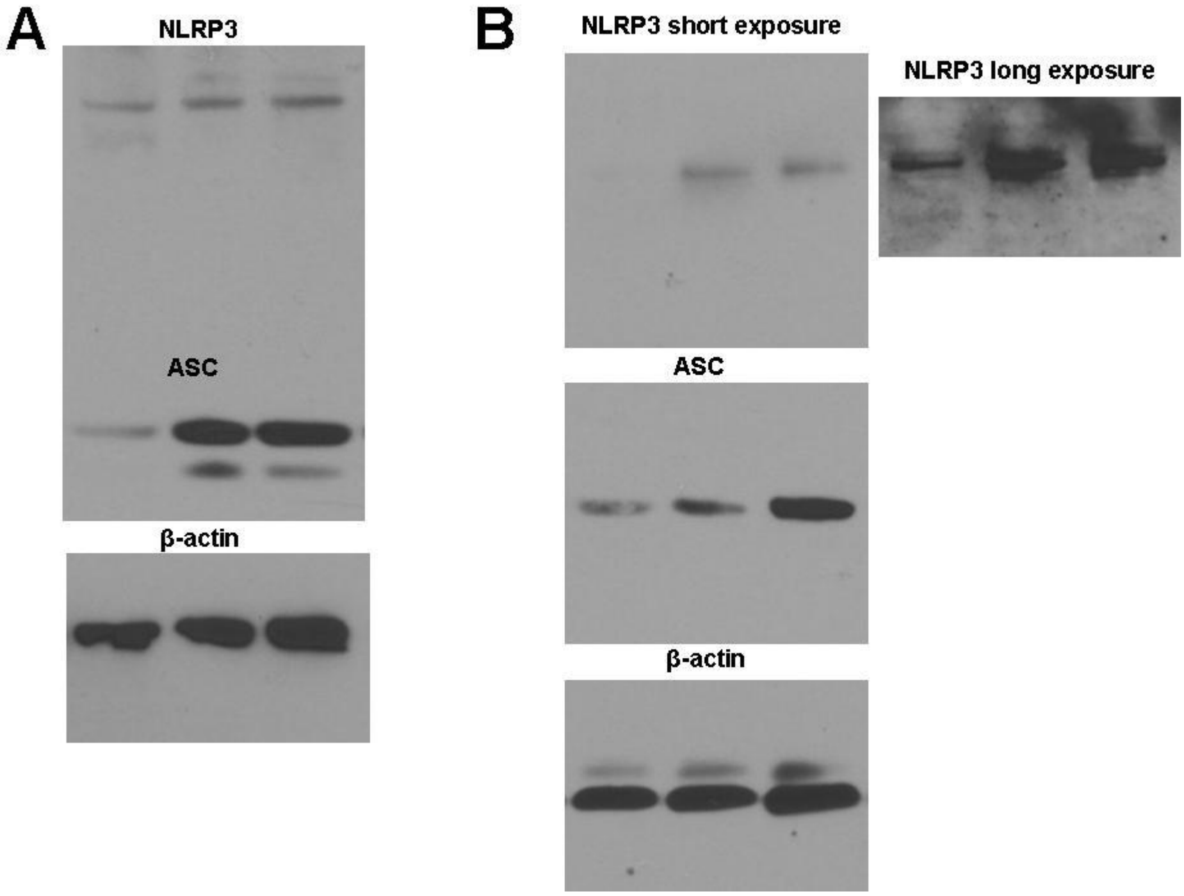

Figure S6. Scans of western blots showing bands representing NLRP3, ASC and β-actin, referred to **(A)** Figure 3E and **(B)** Figure S3B.

Supplementary Methods

***In vivo* model and related measurements**

Mice material from a previous study has been utilized (1); in brief, wild-type C57BL/6 and *Nlrp3* knockout (KO) male mice were fed for 16 weeks ad libitum a control diet (4021.84, AB Diets) or a Western-diet (4021.83, AB Diets); n=8. As previously described (1), urinary creatinine was measured by standardized clinical diagnostic protocol, whereas renal phospholipid and cholesterol content was assessed with kits for quantitative detection from Biolabo.

Preparation of native and oxidized LDL

Human native LDL and LPDS were isolated from plasma (300-400ml) of healthy regular blood donors using sequential KBr-density gradient ultracentrifugation (UC) steps (2).

Briefly, coagulation was induced to eliminate platelets and coagulation factors with addition of $CaCl_2$ (3,675mg/ml) and thromboplastin (Thromborel® S Reagent 1:1000 dilution, Siemens) at 37°C for 30min followed by centrifugation (4000rpm 30min). Very-low density and intermediate-density lipoproteins (VLDL, IDL) were depleted from plasma by UC (58000rpm 22h 4°C, rotor 70Ti, Beckman) with potassium bromide density gradient (density 1,019; KBr gr= Volume plasma ml x 0,019) using Beckman Polyallomer Quick-Seal centrifuge tubes. Top fractions (VLDL and IDL) were discarded and a new density was achieved by adding KBr in the bottom fraction (density 1,063; KBr gr= Volume ml x 0,066). After ultracentrifugation (58000rpm 22h 4°C, rotor 70Ti), the upper fraction (LDL) was harvested and dialyzed (Servapor Dialysis Tubing) against 3L PBS/EDTA (3times, 2h each) and afterwards against 3L PBS (3times, 2h each). The remaining plasma was centrifuged two more times (48000rpm 48h 4°C, rotor 70Ti) for isolation of lipoprotein-deficient serum; after the first UC (density 1,125; KBr gr= Volume ml x 0,095) the HDL-2 upper phase was discarded and the lower part saved for the second UC (density 1,21; KBr gr= Volume ml x 0,136). The bottom LPDS phase was harvested and dialyzed. The protein concentration of the different fractions was determined by Lowry assay and all fractions were sterile filtered.

Mild oxidation of LDL was achieved by dialyzing LDL (1mg protein/ml) against 3L PBS containing 5µM $CuSO_4$ for 36h at 4°C; subsequently oxLDL was dialyzed against PBS/EDTA and PBS. The LDL oxidation in respect to native LDL was verified by agarose gel lipoprotein electrophoresis, precipitation in gel by polyanions and densitometry (Lipidophor® TRIS All In 12, Technoclone).

Healthy plasma donors were selected on basis of the following parameters: blood cholesterol between 60-200mg/dl, HDL-C 35-55mg/dl, LDL-C 30-150mg/dl, lipoprotein A <30mg/dl, triglycerides 20-200 mg/dl, glucose 60-100mg/dl, ApoE3/E3 genotype, BMI <25, age <45 years. Written informed consent and approval from the Ethical Committee (University of Regensburg, Germany) were obtained.

Cell culture and assays procedures

All culture medium were supplemented with 10% foetal calf serum (FCS), 100 IU/ml penicillin, 100 µg/ml streptomycin, 2 mM L-glutamine (Invitrogen). HK2 cells, immortalized murine proximal TEC (IM-PTEC) and primary tubular epithelial cells (isolated as previously described (3)) were cultured in HK2 medium: DMEM/F12 containing 5µg/ml insulin, 5µg/ml transferrin, 5ng/ml sodium selenite, 20ng/ml triiodothyrionine, 50 ng/ml hydrocortisone and 5 ng/ml prostaglandin E1 (Sigma-Aldrich). Primary PTEC were immortalized through expression of the temperature-sensitive mutant tsA58 of the SV40 virus large T antigen under the control of an IFNγ inducible MHC class I promoter; immorto-TEC were grown in HK2 medium with 10ng/ml interferon-ϒ (IFN-ϒ, PROSPEC) at 33°C and differentiated for a week at

37°C without IFN-ϒ (3). MDCK and THP-1 cells were cultured in DMEM and RPMI 1640 medium, respectively.

During metabolic overloading, tubular epithelial cells were culture in 24 or 6-well plates in DMEM medium containing lipoprotein-deficient serum or n/oxLDL (5µg/ml) isolated from healthy plasma donors for 3 or 5 days (4, 5). Medium was replaced every second day. The FA palmitate (Sigma-Aldrich) was complexed with BSA and used at 50µM. In the 3 day-LDL loading assays, drugs were applied at day 0 and day 2: MCC950 (100µM, 1h, AdipoGen), AICAR (20mM, 1h, Enzo Life Sciences), Resveratrol (50µM, 1h, Sigma-Aldrich), SRT1720 (1µM, 30min, Millipore), Z-YVAD-FMK (1µM, continuously, BioVision). U18666A (2,5 /5µg/ml, Sigma-Aldrich), Bafilomycin A1 (12,5 /25Nm, Sigma-Aldrich), phorbol myristoyl acetate (PMA, 2µM, Sigma-Aldrich), Pyocyanin (100µM, Enzo Life Sciences) and tumor necrosis factor-α (TNF-α, 10ng/ml, PROSPEC) were employed as positive control for the induction of phospholipidosis, decrease in lysosomal acidity, increase in cytoplasmic calcium, ROS accumulation and NF-κB activity.

THP-1 cells were differentiated in the presence of 160 ng/ml PMA and subsequently treated with n/oxLDL (40 µg/ml) for 24 h (4, 5).

Gene silencing and transient ectopic gene expression

For generation of stable knockdown HK2 cells, cells were transduced with lentiviral particles harbouring the sh/sgRNA in presence of 8µg/ml polybrene (Sigma Aldrich) for 24h and after 48h transduced cells were selected with 10µg/ml puromycin (Sigma Aldrich). Lentiviral particles were produced by transfecting HEK293T cells, using GENIUS DNA Transfection Reagent (Westburg), with pMD2.G/VSVG (Addgene 12259), pPAX2 (Addgene 12260) and the lentiviral sh/sgRNA vector (6:15:20 µg DNA ratio). Lentiviral pLKO.1 vectors (Addgene 10878, (6)) containing an shRNA insert targeting human (h) NLRP3 (TRCN0000062725), PYCARD/ASC (TRCN0000059074), CASP1 (TRCN0000003503) and SIRT1 (TRCN0000018979) were used. Single guide RNA targeting NLRP3, ASC and CASP1 were inserted in pLentiCRISPRv2 (Addgene 52961) following the protocol of Sanjana *et al.* (7). Targeting sequence were GAAGAAGACGTACACCGCGG for hNLRP3, TCTTGAGCTCCTCGGCGGTC for hASC, GCTAACGTGCTGCGCGACAT for hCASP1. For transient overexpression of Sirtuin-1, HK2 cells were transfected with pSIRT1 Flag (Addgene 13812) using GENIUS DNA Transfection Reagent (Westburg).

Flow cytometric analysis

To assess phospholipid accumulation, the HCS LipidTOX™ Red phospholipidosis detection reagent (Thermo Fisher Scientific) was diluted 2000 times in stimulation medium 24h prior measurement. For the detection of alterations of the endolysosomal compartment, 50nM LysoTracker Red DND-99 (Thermo Fisher Scientific) and 1µM pH-sensitive LysoSensor Green DND-189 (Thermo Fisher Scientific) were added to alive cells for 1h. HK2 cells were loaded with 50µg/ml FITC-Dextran 10/40 KDa (Sigma-Aldrich) 90min following 3 days LPDS/LDL exposure. The Fluo-4-AM (Invitrogen) green fluorescence calcium indicator was added to alive stimulated cells at 1µM 20 min prior harvesting; accordingly to the manufacturer's instructions longer incubation would stain calcium storage organelles. Mitochondria alterations were detected using the MITO-ID® Membrane Potential Detection Kit (green/orange fluorescence) and the green fluorescent Oxidative Stress Detection Reagent (Enzo Life Sciences) following the manufacter's instructions. The green fluorescent glucose analogue 2-NBDG (5mM, Cayman Chemical) has been used to monitor glucose uptake in live MDCK cells after 1h incubation. The fluorescent inhibitor probe FAM-YVAD-FMK (ImmunoChemistry Technologies) was adopted to label active caspase-1 enzyme in living HK2 cells after 3 days of metabolic loading.

Staining was visualized on FACSCalibur, FACSCanto II or LSRFortessa (BD Biosciences) and analysis was done using FlowJo software (TreeStar).

Immunofluorescence and immunohistochemistry staining

Phospholipidosis was visualized by adding HCS LipidTOX™ Red phospholipidosis detection reagent to cells cultured on 12mm glass coverslips. Nuclei were stained with 0,5µg/ml DAPI (Sigma-Aldrich) after cell fixation with 4% paraformaldehyde (PFA) in PBS. Coverslips were mounted on glass with Vectashield Mounting Media (Vector Laboratories).

Active caspase-1 was stained in alive cells with the FAM-YVAD-FMK fluorescent probe (ImmunoChemistry Technologies); after cell fixation with 4% PFA-PBS, nuclei were stained with 0,5µg/ml DAPI. ASC was detected by anti-ASC (Enzo Life Sciences) staining followed by anti-rabbit IgG Alexa Fluor 488 (Invitrogen).

For imagining of lysosomal leakage, cells were loaded with FITC-Dextran 40KDa (Sigma-Aldrich) for 90 min and lysosomes were labelled with LysoTracker Red DND-99 (Thermo Fisher Scientific).

Frozen sections from kidney biopsies (patients with obesity and hypercholesterolemia or with diabesity or control biopsy) were 1% glutaraldehyde and stained for phospholipid for 15 min with Nile Red (0,1µg/ml in PBS; Sigma Aldrich); nail polish was employed for sealing the cover slides to the glasses. Immunofluorescence stainings were visualized with a Leica DM5000B microscope (Leica-microsystems).

Sirtuin-1 was visualized on paraffin sections from murine kidneys with DAB substrate using peroxidase-based detection after consequential staining with anti-SIRT1 (Abcam) and anti-mouse IgG1 HRP-conjugated (DAKO). Heat-mediated antigen retrieval with sodium citrate was performed prior to staining (3).

LDH and ATP detection assays

LDH released from damaged cells was measured in the cell supernatant with the LDH-Citotoxicity Assay Kit (BioVision). Bioluminescent detection of cellular ATP was done using the ViaLight™ Plus BioAssay Kit (Millipore).

NF-κB reporter

NF-κB signaling activation was assessed by luciferase activity assay. Cells seeded in a 24-well plate were concomitantly transfected with NF-κB luciferase reporter (0,666µg/well, Addgene) and pRL-TK-Renilla (0,333µg/well, Addgene) using GENIUS DNA Transfection Reagent (Westburg). Firefly and Renilla luciferase activities were measured by Dual-Luciferase® Reporter Assay System (Promega) on a GloMax®-Multi Detection System (Promega). NF-κB activity is indicated by the ratio in luminescence units from NF-κB-driven Firefly luciferase/ control Renilla luciferase.

ELISA

Specific ELISAs (R&D Systems) were utilized to measure IL-1β and IL-6 in cell supernatant according to the manufacter's instructions.

Quantitative Real-time PCR

Total RNA was extracted from 10 frozen renal sections (30µm thick) or from cells with Trizol reagent (Invitrogen). RNA was converted to cDNA by using oligo-dT primers. Quantitative real-time PCR (Q–PCR) was performed on a LightCycler® 480 System (Roche) using SYBR Green-SensiMix (Bioline). SYBR green dye intensity was analyzed with linear regression analysis. Gene-expression was normalized towards the housekeeping genes TATA-box binding protein (TBP), glyceraldehyde 3-phosphate dehydrogenase (GAPDH) and hypoxanthine guanine phosphoribosyltransferase (HPRT).

Western blotting

Cells were incubated at 4°C for 30 minutes in RIPA buffer containing 20mM Tris-HCl pH7.5, 150mM NaCl, 5mM EDTA, 1% NaDOC, 1% NP-40, 200µM Na_3VO_4, 50mM NaF, 1% protease inhibitor cocktail (P8340, Sigma), 0.1% SDS, 10% glycerol.

Cell lysates were subjected to Western blotting using anti-LAMP-2-FITC (LifeSpan BioSciences), anti-SIRT1 (Abcam), anti-phospho- and total AMPK, LKB1 (Cell Signaling Technologies). HRP-conjugated secondary antibodies (DAKO) were used and HRP activity was visualized with peroxidase substrate for enhanced chemiluminescence (ECL Western Blotting Substrate, Thermo Scientific). β-actin (Abcam) were used as loading controls for cytoplasmic and nuclear fractions, respectively. Densitometric quantification analysis was performed on imagines of scanned films using the Image J software.

Lipidomics

Lipids were isolated from homogenized renal tissues with the chloroform/methanol extraction procedure described by Bligh and Dyer (8) in the presence of not naturally occurring lipid species as internal standards; the chloroform phase was dried in a vacuum centrifuge. Lipids were quantified by direct flow injection electrospray ionization tandem mass spectrometry (ESI-MS/MS) in positive ion mode, using the analytical setup and strategy described previously (9). Sphingolipids were extracted by the butanolic extraction procedure described by Baker *et al*. (10, 11). The butanol phase was evaporated to dryness under reduced pressure. Sphingolipid analysis was done using a previously established liquid chromatography-tandem mass spectrometry (LC-MS/MS) protocol (12, 13).

Quantification was achieved using non-naturally occurring internal standards and calibration lines generated by standard addition of a number of naturally occurring species to samples. Self-programmed Excel Macros were employed for deisotoping and data analysis of all lipid classes (9, 12).

Biochemical analysis

Urine electrolytes were measured using standardized clinical diagnostic protocols of the Academical Medical Center Amsterdam.

Statistics

Statistical analysis was performed using One-Way ANOVA and Dunnett's tests; Mann-Whitney U test (Student t test used for analysis of data shown in Figure S2) was used for two group comparison and Spearman rank correlation to test the association between two ranked variables. In the dotplot graphs, each dot represents the average of one independent experiment. Mean and standard error of the mean (SEM) are indicated for each condition; $P < 0.05$ was considered to be significant.

References

1. Bakker PJ *et al.* Nlrp3 is a key modulator of diet-induced nephropathy and renal cholesterol accumulation. *Kidney Int.* 85(5), 1112-22 (2014).
2. Orso E, Matysik S, Grandl M, Liebisch G & Schmitz G. Human native, enzymatically modified and oxidized low density lipoproteins show different lipidomic pattern. *Biochim Biophys Acta.* 1851(3), 299-306 (2015).
3. Rampanelli E *et al*. Opposite role of CD44-standard and CD44-variant-3 in tubular injury and development of renal fibrosis during chronic obstructive nephropathy. *Kidney Int.* 86(3), 558-69 (2014).
4. Grandl M & Schmitz G. Fluorescent high-content imaging allows the discrimination and quantitation of E-LDL-induced lipid droplets and Ox-LDL-generated phospholipidosis in human macrophages. Cytometry A. 77(3), 231-42 (2010).
5. Grandl M *et al.* E-LDL and Ox-LDL differentially regulate ceramide and cholesterol raft microdomains in human Macrophages. Cytometry A. 69(3), 189-91 (2006).

6. Moffat J *et al.* A lentiviral RNAi library for human and mouse genes applied to an arrayed viral high-content screen. *Cell.* 124(6), 1283-98 (2006).
7. Sanjana NE, Shalem O & Zhang F. Improved vectors and genome-wide libraries for CRISPR screening. *Nat Methods.* 11(8), 783-4 (2014).
8. Bligh EG & Dyer WJ. A rapid method of total lipid extraction and purification. *Can J Biochem Physiol.* 37(8), 911-7 (1959).
9. Liebisch G *et al*. High throughput quantification of cholesterol and cholesteryl ester by electrospray ionization tandem mass spectrometry (ESI-MS/MS). *Biochim Biophys Acta.* 1761(1), 121-8 (2006).
10. Baker DL, Desiderio DM, Miller DD, Tolley B & Tigyi GJ. Direct quantitative analysis of lysophosphatidic acid molecular species by stable isotope dilution electrospray ionization liquid chromatography-mass spectrometry. *Anal Biochem.* 292(2), 287-95 (2001).
11. Scherer M, Schmitz G & Liebisch G. High-throughput analysis of sphingosine 1-phosphate, sphinganine 1-phosphate, and lysophosphatidic acid in plasma samples by liquid chromatography-tandem mass spectrometry. *Clin Chem.* 55(6), 1218-22 (2009).
12. Scherer M, Schmitz G & Liebisch G. Simultaneous quantification of cardiolipin, bis(monoacylglycero)phosphate and their precursors by hydrophilic interaction LC-MS/MS including correction of isotopic overlap. *Anal Chem.* 82(21), 8794-9 (2010).
13. Scherer M, Bottcher A, Schmitz G & Liebisch G. Sphingolipid profiling of human plasma and FPLC-separated lipoprotein fractions by hydrophilic interaction chromatography tandem mass spectrometry. *Biochim Biophys Acta.* 1811(2), 68-75 (2011).
